# Daily Objective Physical Activity and Sedentary Time in Adults with COPD Using Spirometry Data from Canadian Measures Health Survey

**DOI:** 10.1155/2018/9107435

**Published:** 2018-12-02

**Authors:** P. Bernard, G. Hains-Monfette, S. Atoui, G. Moullec

**Affiliations:** ^1^Department of Physical Activity Sciences, Université du Québec à Montréal, Montréal, QC, Canada; ^2^Research Center, University Institute of Mental Health at Montreal, Montréal, QC, Canada; ^3^Department of Social and Preventive Medicine, School of Public Health, University of Montréal, Montréal, QC, Canada; ^4^Research Center, CIUSSS Nord-de-l'Île-de-Montréal, QC, Canada

## Abstract

Chronic obstructive pulmonary disease (COPD) is expected to be the third leading cause of premature death and disability in Canada and around the world by the year 2020. The study aims to compare objective physical activity (PA) and sedentary time in a population-based sample of adults with chronic obstructive pulmonary disease (COPD) and compare a group, and to investigate whether these behaviors differ according to COPD severity. From the 2007–2013 Canadian Health Measures Survey dataset, accelerometer and prebronchodilator spirometry data were available for 6441 participants, aged 35 to 79. Two weighted analyses of covariance were performed with adjustments for age, sex, body mass index, accelerometer wearing time, season, work, smoking (cotinine), education level, and income. A set of sensitivity analyses were carried out to examine the possible effect of COPD and type of control group. A cross-sectional weighted analysis indicated that 14.6% of study participants had a measured airflow obstruction consistent with COPD. Time in PA (moderate-vigorous and light PA), number of steps, and sedentary duration were not significantly different in participants with COPD, taken together, compared to controls. However, moderate to severe COPD participants (stages ≥2) had a significantly lower daily time spent in PA of moderate and vigorous intensity level compared to controls. Canadian adults with COPD with all disease severity levels combined did not show lower daily duration of light, moderate, and vigorous PA, and number of steps and higher daily sedentary time than those without airflow obstruction. Both groups are extremely sedentary and have low PA duration. Thus, “move more and sit less” public health strategy could equally target adults with or without COPD.

## 1. Introduction

Chronic obstructive pulmonary disease (COPD) is a public health issue in Canada [1]. Indeed, COPD is a leading cause of disability, morbidity, and mortality. COPD is among the most common causes of hospitalization and health care utilization in Canadian adults [2, 3]. Comorbidities are highly prevalent in people with COPD [4] increasing the risk of physical inactivity. Lack of physical activity and majority of daily time spent in sedentary behaviors have been independently associated with high mortality rates in adults with COPD [5, 6].

Physical activity and sedentary are complex behaviors in adults with COPD. Patterns of physical activity and sedentariness have been previously examined in COPD population using self-reported—as opposed to objective—measures [7, 8]. Previous cross-sectional investigations established that adults with COPD have a significantly reduced duration of moderate and vigorous physical activity (MVPA), light physical activity (LPA), and number of steps when compared to age-matched controls [9–11]. Previous studies also compared the daily steps in COPD outpatients and controls. COPD patients performed significantly fewer steps [10, 12, 13]. In the last systematic review focusing on objectively assessed daily physical activity comparison in adults with COPD and control group, Vorrink et al. concluded that adults with COPD have a significantly reduced duration, intensity, and frequency of physical activity [11]. They suggested that these differences may be due to possible selection bias (i.e., recruitment of more severe patients from rehabilitation programs and recruitment of highly physical active controls (seniors involved in a physical fitness/exercise group) [11, 14]. However, a recent population-based study compared accelerometer data in adults with self-reported COPD diagnosis to controls, using the National Health and Nutrition Evaluation Survey (NHANES) data, a representative US national sample. People with COPD were less active and spent more time in sedentary behaviors. However, the self-reported COPD status was an important limitation due to its frequent underdiagnosis in the general population [15, 16]. To date, a comparison of objectively measured physical activity and sedentary behaviors data between adults with or without spirometry-based classification of COPD remains to be done, across severity stages in a population-based study.

We aimed to compare objectively measured physical activity and sedentary behaviors in a representative sample of Canadian adults with measured airflow obstruction consistent with COPD (ratio of forced expiratory volume in 1 second to the forced vital capacity less than 0.70) with a control group with no COPD. We also investigated how physical activity and sedentary behaviors differ according to COPD severity.

## 2. Methods

### 2.1. Study Population

This study used data collected during cycles 1, 2, and 3 (2007–2013) of the Canadian Health Measures Survey (CHMS; representative of approximately 96% of the Canadian population) [17]. Data were collected in two stages. First, sociodemographic and general health information were collected during an in-person interview at the participants' homes. Then, direct physical measurements (i.e., cotinine level, weight, height, and spirometry) were collected by trained health technicians during a subsequent visit to a mobile examination center. We included participants aged 35 to 79 years with a spirometry test and complete physical activity data. Ethical approval to conduct the survey was obtained from Health Canada's Research Ethics Board [17]. All respondents provided written informed consent.

### 2.2. Measures

#### 2.2.1. Sociodemographic and Clinical Characteristics

Age, level of education, household income, working status (yes/no for last 12 months), marital status, and body mass index were reported as sociodemographic characteristics. Smoking was characterized with the following variables: age of first cigarette smoked, daily cigarette consumption, number of daily smoking years, and urinary cotinine level. Cotinine provided an accurate quantitative measure of active or second-hand smoking. Participants were asked to refrain from smoking for a 2-hr period prior to the mobile-examination-clinic visit [18]. The following self-reported information was documented with yes/no questions: self-reported diagnosis of COPD, cough, phlegm, shortness of breath, and mood disorders. Participants reported their sleep duration and used a Likert scale ranging from *never* to *all the time* to assess their sleep problems, restorative sleep, and difficulty to stay awake. Self-reported physical health, mental health, perceived stress, and quality of life were each evaluated with one item, using a response scale ranging from *poor* to *excellent*.

### 2.3. Physical Activity

Upon completion of their mobile examination center visit, CHMS participants were asked to wear an Actical accelerometer (Philips-Respironics, 17 grams, omnidirectional accelerometer) [19] over their right hip on an elasticized belt during their waking hours for 7 consecutive days. The Actical began measuring the day following the mobile examination center appointment. The accelerometers were returned by post mail to Statistics Canada. Then, the monitor was checked to determine if it was still within the manufacturer's calibration specifications. The Actical is a valid accelerometer to assess physical activity in adults [19, 20]. It measures and records time-stamped acceleration in all directions, thereby indicating the intensity of physical activity. The digitized values are summed over a user-specified interval of 1 minute, resulting in a count value per minute (cpm) and steps accumulated per minute [21]. All data were blind to respondents while they were wearing the device. As recommended for adults with COPD [22], a valid day was defined as 8 or more hours of wear time; respondents with 4 or more valid days were retained for analyses (FEV_1_/FVC values between participants with valid and no valid actimeter data were compared). Participants with extreme counts (i.e., >20,000 cpm) were excluded from analyses [23]. The daily time spent in physical activity of different intensity levels was categorized using validated cpm thresholds for adults [24]: sedentary (<100 cpm), light (100 to 1534 cpm), and moderate to vigorous (≥1535 cpm).

### 2.4. Prebronchodilator Spirometry Measures

The prebronchodilator spirometry procedure followed the revised joint American Thoracic Society/European Respiratory Society guidelines [25]. Spirometry was performed with a modern Fleisch pneumotachograph-type spirometer (KoKo; nSpire Health, Longmont, CO). The spirometer was always the same for all CHMS participants. Technician training for the mobile examination center was the same for all operators, and quality control assessment was done both manually and electronically. The spirograms were saved, and all tracings were reviewed at a later date by a qualified pulmonary function technician who made the final acceptance or rejection of tracings from the field based on previously agreed-on criteria. The forced vital capacity (FVC), forced expiratory volume in one second (FEV_1_), and their ratio (FEV_1_/FVC) were collected. Predicted values for these lung function parameters were derived from prediction equations from the Third NHANES [26]. The Global Initiative for Obstructive Lung Disease (GOLD) criteria for prebronchodilator data were used to identify COPD cases (FEV_1_/FVC < 0.70) and to determine severity stages [27].

For each participant, the measured FEV_1_/FVC was calculated from the largest set of FEV_1_ and FVC values recorded in any of the spirometric maneuvers for which participant performance met American Thoracic Society acceptability criteria. Respondents were not eligible to perform spirometry if they were pregnant for more than 27 weeks, had a heart attack or major surgery in the chest or abdomen in the previous 3 months, had eye surgery in the previous 6 weeks, reported taking medication for tuberculosis, or had an acute respiratory tract infection (e.g., cold, flu, and so on) [28] or other conditions that would likely make spirometry unsafe [29].

### 2.5. Statistical Analysis

To account for the complex, multistage probability sampling design, weights (i.e., activity monitor subsample weights combining cycles 1, 2, and 3) and bootstraps provided by the CHMS were used in the analyses. All analyses were performed during 2017 and 2018, using *survey* [30] package in R version 3.3. A set of weighted analyses of covariance regression models (ANCOVA) incorporating age, sex, body mass index, accelerometer wearing time, season, work, smoking (cotinine), education level, and income were carried out. As suggested by Aaron et al. [31], in order to limit a possible COPD misclassification, the absolute distance of the participant's FEV_1_/FVC ratio from 0.70 was also included in our models. Different models were tested for the following outcomes: average steps per day, average minutes per day of LPA and MVPA, and average minutes per day of sedentary behavior. Since the number of minutes of MVPA was not normally distributed, Poisson models were carried out for this variable.

### 2.6. Sensitivity Analyses

To examine the possible effect of COPD characterization, type of control group, and poor adherence to accelerometer, a set of sensitivity analyses was planned. A more conservative definition of airflow limitation defined as FEV_1_/FVC less than the lower limit of normal (LLN), using the Canadian references from CHMS [28, 32], was used. Then, ANCOVAs were carried out with physical activity and sedentary outcomes. We also performed analyses by excluding controls with chronic diseases (i.e., diabetes, cancer, heart disease, and fibromyalgia).

## 3. Results

### 3.1. Descriptives

In total, 6,441 participants were included. Cross-sectional weighted analyses indicated that 14.6% of CHMS participants aged 35 to 79 had a measured airflow obstruction consistent with COPD and 1.3% self-reported a COPD diagnosis. Among these COPD cases, 49.3% were classified as GOLD stage 1, 45% stage 2, and 5.7% stages 3-4. The average age were 56.9 (0.7), 57.2 (0.9), and 59.9 (3.4) in participants classified in GOLD stages 1, 2, and 3-4, respectively. For the comparison group, the average age was 52.2 (SE = 0.2) years and 48% were male. On average, participants with COPD spent 16.5 (SE = 1), 216 (SE = 5), and 447 (SE = 11) minutes per day engaged in MVPA, LPA, and sedentary time, respectively. The detailed participant characteristics are shown in Tables 1 and 2 and Supplementary file 1. The FEV1/FVC values between participants with (*N* = 6,641) and without (*N* = 606) valid accelerometer data were not significantly different (*p*=0.15).

### 3.2. Accelerometry-Measured Physical Activity and Sedentary Time in Participants with COPD

**Figure 1:**
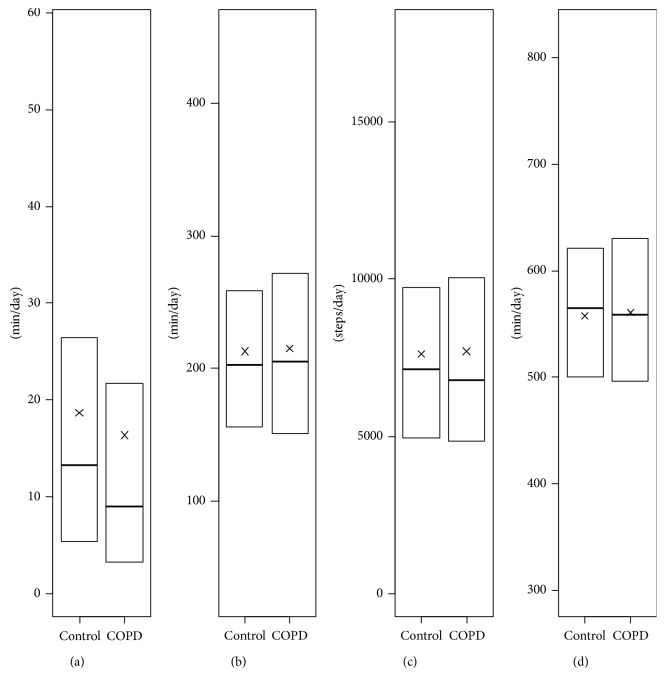
Daily physical activity and sedentary in participants with and without COPD. (a) Moderate and vigorous physical activity (MVPA). (b) Light physical activity (LPA). (c) Steps. (d) Sedentary. x = mean. The whiskers are voluntary missing because Statistics Canada does not allow figures with individual data representation.

**Figure 2:**
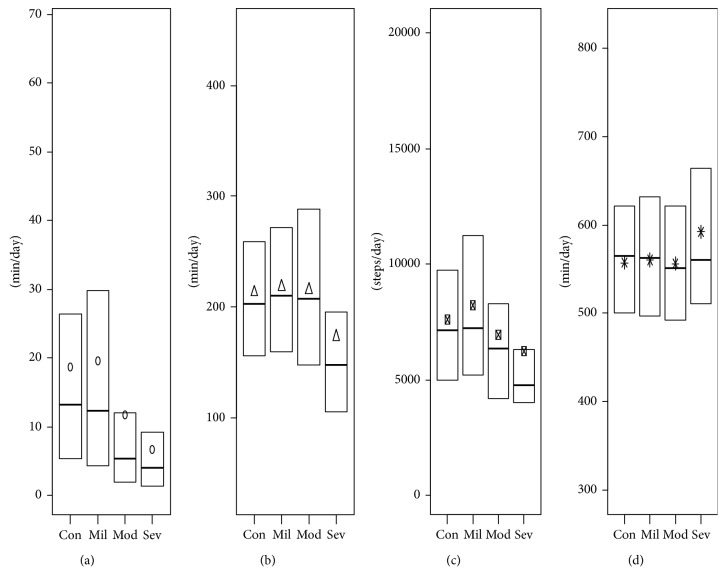
Physical activity and sedentary levels between control participants and COPD severity. (a) Moderate and vigorous physical activity (MVPA). (b) Light physical activity (LPA). (c) Steps. (d) Sedentary. Con = control, Mil = COPD mild severity, Mod = COPD moderate severity, Sev = COPD (very) severe. The whiskers are voluntary missing because Statistics Canada does not allow figures with individual representation.

We found that physical activity and sedentary duration were not significantly different in participants with COPD, taken all together, compared to controls (Figure 1). Participants classified in stages 2 and 3-4 had a significant lower daily MVPA duration in comparison to controls (11.7 min and 6.7 min versus 18.7 min; *p* < 0.01; effect size (ES) = 0.14 and ES = 0.54, respectively) (Table 3). No other significant differences were observed across COPD severity (Figure 2). All details about statistical results are available in Supplementary files 2 and 3.

### 3.3. Sensitivity Analyses

In sensitivity analyses, using a “healthy” control group (i.e., excluding participants with chronic diseases, *N* = 4312), similar results were found for physical activity and sedentary outcomes (Table 2). When we used the COPD case definition as FEV_1_/FVC<LLN, leading to a weighted prevalence of 9.6%, no significant differences were observed for MVPA, LPA, steps, and sedentary variables. Descriptive results and findings from sensitivity analyses are presented in Supplementary files 2 and 3.

## 4. Discussion

Previous studies conducted in convenient samples have shown that COPD patients were less active and spent more time in sedentary behaviors than their same-age peers [10, 11, 33]. This population-based study, with spirometry-based characterization, is the first one to robustly show that COPD patients (taken all together) spend equivalent daily durations of physical activity and sedentary time when compared to age-matched controls. This inconsistency may be due to recruitment strategies in previous studies where COPD patients were mostly recruited from rehabilitation programs [11, 33] or cancer screening trial [10], and their peer controls were active seniors [34] or students [35]. For instance, the daily number of steps in control groups was around 7620 and 9400 in the current study and in Trooster's investigation [12], respectively. Consequently, previous studies may have included more severe COPD patients. Another possible explanation is the important difference in the prevalence of COPD, confirmed by spirometry (14.6%) or self-reported (1.3%) [27], among CHMS participants. Consequently, a substantial part of CHMS participants seems to be undiagnosed. Yet, using the NHANES cohort, Martinez and colleagues [36] demonstrated that patients with undiagnosed COPD had better lung function and health status compared to those with physician-diagnosed COPD. As previously shown [37, 38], this may explain why CHMS participants with undiagnosed COPD (13.4%) reported higher physical activity levels. This inconsistency may be explained, at least in part, by the fact that the classification of participants with mild-to-moderate airflow obstruction based only on spirometry data is associated with higher risk of misclassification [31].

Our results support that daily sedentary duration was not related to COPD status or severity. This finding is consistent with previous evidence showing an equivalent daily time spent among adults with self-reported COPD and controls [16]. Regarding physical activity across COPD severity, only daily time spent in MVPA was significantly lower in participants in the later stages of COPD. The lack of differences in LPA duration and number of steps across severity stages is consistent with previous data [33]. These results also suggest a floor effect due to aging, where most of the Canadian elderly have adopted a lifestyle including few MVPA [39]. COPD mainly affects LPA in everyday life and leisure, which is likely to play an important role in the deterioration of patient's quality of life [40].

While findings suggest that there is a modest impact of COPD on these behaviors, the promotion of physical activity and prevention of prolonged sedentary time by clinicians should not be discouraged. Physical activities of light intensity should be considered as a priority target in the most advanced COPD patients. Indeed, physical activity and sedentary behaviors, objectively assessed, are among the strongest predictors of mortality in adults with COPD [5, 6].

### 4.1. Strengths and Limitations

This is the first time that data from a representative national sample of adults have been used to compare objectively measured physical activity and sedentary variables between adults with COPD (confirmed with spirometry data) and age-matched controls. However, the limitations of this study need to be considered when interpreting the results. First, a misclassification bias of COPD cases cannot be ruled out because only prebronchodilatator spirometry data were available in CHMS. The prevalence of measured airflow obstruction was two times higher than self-reported diagnosis of COPD [27]. However, this prevalence was consistent with those found in North America [41]. Second, the CHMS dataset contained no validated indicator of anxiety or depression. Yet, these parameters are known to affect the physical activity and sedentary behaviors in adults with COPD [42]. Third, the cross-sectional design did not allow inference on the evolution of physical activity across COPD severity stages.

## 5. Conclusions

In conclusion, participants with COPD did not perform a lower daily duration of MVPA and LPA, number of steps, and higher daily sedentary time than those without airflow obstruction in Canada. The daily time spent in physical activity of moderate and vigorous intensity is impaired in severe COPD adults. Our results highlight that both groups are extremely sedentary and have low duration of MVPA and LPA. Thus, a “move more and sit less” public health strategy [43], arguing in favor of interventions targeting physical activity and sedentary behaviors could equally target adults with or without COPD.

## Figures and Tables

**Table 1:** Weighted sample characteristics for adults with airflow obstruction consistent with COPD and the comparison group.

	Control	COPD	Univariate test	*p*
Sex (men), %*N*	47.8 (7,209,724)	55.2 (1,437,262)	*F* = 4.8	0.03

Age (years), M (Se)	52.2 (0.2)	57.1 (0.5)	*t* = 7.7	0.0001^e−09^

BMI, M (Se)	27 (0.2)	25.77 (0.3)	*t* = −5.1	0.0004^e−03^

*Worked at job last year*, *%(N)*				
Yes	73.8 (11,120,963)	59.6 (1,548,769)	*F* = 17.1	0.0007^e−03^
No	23.6 (3,554,449)	34.2 (888,519)		
Study/retired	2.7 (400,151)	6.2 (161,012)		

Marital status (alone), %(*N*)	23.4 (3,521,001)	35.3 (921,516)	*F* = 14.8	0.0001

*Income*, *%(N)*				
<$15k	3.3 (503,026)	5.7 (148,953)	*F* = 4	0.0004
$15k–$19.99k	2.1 (314,984)	4.7 (122,368)		
$20k–$29.99k	7.6 (1,145,997)	11.7 (305,177)		
$30k–$39.99k	9.3 (1,406,009)	13.8 (360,495)		
$40k–$49.99k	10 (1,510,219)	10.2 (266,676)		
$50k–$59.99k	8.3 (1,258,115)	9.7 (252,317)		
$60k–$79.99k	15.5 (2,336,038)	13 (338,775)		
$80k-$99.99k	12.1 (1,825,625)	8.8 (228,037)		
≥$100k	31.6 (4,768,563)	22.4 (582,489)		

*Education*, *%(N)*				
Lower than high school	4.5 (684,534)	3.7 (95 553)	*F* = 6.1	0.0001^e−01^
High school	8.7 (1,320,078)	16.7 (430,033)		
Work school	17 (2,560,089)	16.1 (414,645)		
College	9.6 (1,456,458)	6.3 (163,051)		
University < bachelor	12.3 (1,861,439)	7.8 (200,974)		
University ≥ bachelor	17.2 (2,591,464)	11.9 (304,806)		
Missing	30.7 (4,629,193)	37.4 (961,546)		

*Self-reported symptoms and disease*				
Self-reported COPD, %(*N*)	0.3 (48 394)	6.6 (172,681)	*F* = 106.2	0.0002^e−12^
Cough phlegm regularly, %(*N*)	9.9 (1,493,964)	20.4 (531,364)	*F* = 34.7	0.0007^e−05^
Simple chores make short of breath, %(*N*)	9.1 (1,374,510)	17.9 (466,106)	*F* = 22.2	0.0003^e−02^
Self-rated health, %(*N*)				
** **Fair/poor	11.2 (1,687,499)	16.6 (433,036)	*F* = 8.1	0.005
** **(Very)good/excellent	88.8 (13,381,077)	83.4 (2,172,250)		
Self-rated health compared to 1 year ago, %(*N*)				
** **Much better/somewhat/about the same	86.6 (13,043,443)	83.4 (2,172,889)	*F* = 1.7	0.19
** **Somewhat/much worse	13.4 (2,025,133)	16.6 (432,396)		
Self-reported mood disorder, %(*N*)	11 (1,653,201)	11.9 (309,163)	*F* = 0.2	0.64
Other physical or mental conditions, %(*N*)	20.7 (3,123,929)	17.2 (447,954)	*F* = 1.8	0.18

*Psychosocial outcomes*				
Self-rated quality of life, %(*N*)				
** **Fair/poor	6.1 (915,796)	6.7 (175,346)	*F* = 0.1	0.72
** **(Very)good/excellent	93.9 (14,158,701)	93.3 (2,424,018)		
Self-rated mental health, %(*N*)				
** **Fair/poor	5.4 (818,961)	7.5 (195,594)	*F* = 1.1	0.3
** **(Very)good/excellent	94.6 (14,256,256)	92.5 (2,403,050)		
Self-rated stress, %(*N*)				
** **Not at all/not very/a bit stressful	78.4 (11,813,678)	78.3 (2,043,899)	*F* = 0.002	0.97
** **Quite a bit/extremely stressful	21.6 (3,249,461)	21.7 (566,824)		

*Sleep outcomes*				
Sleep duration, M (Se)	7 (0.03)	7.1 (0.06)	*t* = 0.6	0.54
Frequency of sleep problems, %(*N*)				
** **Never/rarely/sometimes	77.8 (11,712,338)	74.2 (1,940,200)	*F* = 2.59	0.06
** **Most of the/all the time	22.2 (3,347,745)	25.8 (673,579)		
Restorative sleep, %(*N*)				
** **Never/rarely/sometimes	40.7 (6,127,211)	37.6 (982,096)	*F* = 1.9	0.14
** **Most of the/all the time	59.3 (8,932,682)	62.4 (1,631,872)		
Difficulty staying awake, %(*N*)				
** **Never/rarely/sometimes	95 (14,316,265)	94.7 (2,473,213)	*F* = 0.03	0.86
** **Most of the/all the time	5 (746,161)	5.3 (138,222)		
*Smoking variables*				
Smoking, %(*N*)	16.1 (2,428,599)	43 (1,121,876)	*F* = 86.4	0.0002^e−12^
Age at first smoking the whole cig, M (Se)	16 (0.2)	15.5 (0.2)	*t* = −1.8	0.07
Age at smoking everyday, M (Se)	18.9 (0.2)	19 (0.8)	*t* = 0.1	0.95
Number of cig smoked/day when at least one cig/month, M (Se)	6.1 (0.2)	6.7 (0.3)	*t* = 1.5	0.13
Number of years smoked daily (former daily smokers), M (Se)	18.3 (0.5)	24 (1)	*t* = 5.2	0.000^3e−03^
Levels of cotinine, M (Se)	215.2 (19.1)	609.9 (60.1)	*t* = 6.2	0.0001^e−05^

*Spirometry*				
FEV_1_/FVC, M (Se)	0.78 (0.001)	0.64 (0.003)	*t* = −46.6	0.0002^e−12^
FEV_1_ predicted, M (Se)	103.5 (0.4)	84.9 (0.9)	*t* = −18.8	0.0002^e−12^
*Characteristics of physical activity and sedentary*				

Acc wearing time (min/day), M (Se)	13.7 (0.04)	13.8 (0.1)	*t* = 0.5	0.63
MVPA (min/day), M (Se)	18.7 (0.6)	16.4 (1.5)	*t* = −1.9	0.06
LPA (min/day), M (Se)	213.1 (2.8)	215.2 (5.6)	*t* = 0.4	0.71
Steps (steps/day), M (Se)	7621.2 (111.9)	7712.7 (256.5)	*t* = 0.3	0.74
Sed (min/day), M (Se)	557.3 (2.4)	560.6 (6.6)	*t* = 0.4	0.66

*Note.* BMI = body mass index, cig = cigarette, Acc = accelerometer, LPA = light physical activity, MVPA = moderate and vigorous physical activity, Sed = sedentary behavior.

**Table 2:** Weighted characteristics of physical activity and sedentary.

	MVPA (min/day)				LPA (min/day)				Steps (steps/day)				Sed (min/day)			
	M	Se	Md	IQR	M	Se	Md	IQR	M	Se	Md	IQR	M	Se	Md	IQR
Control	18.7	0.6	13.3	21	213.1	2.8	203	103	7621	111.9	7148.6	4758.7	557.3	2.4	564.5	120.7
Healthy control	20	0.7	14.6	22	219.5	2.9	208.5	104.2	7897	119.1	7418.9	4624.8	554.8	2.5	562.3	123
COPD	16.4	1.5	9	18.4	215.2	5.6	205.2	121.1	7713	256.5	6792.9	5194.2	560.6	6.6	558.6	133.5
GOLD stage I	19.6	1.8	12.3	25.5	218.6	6.3	210.7	112.4	8228	294.5	7232.1	6010.8	560.4	8.9	562.2	135.4
GOLD stage II	11.7	1.5	5.4	10	215.2	8.3	207.2	140	6953	335.4	6364.3	4089.4	556.1	8.8	550.7	129.2
GOLD stage ≥ III	6.7	1.5	4.1	7.8	172.9	23	147.7	90.3	6240	876.5	4782.1	2293.2	592.2	28.4	560.8	153.9

*Note.* M = mean, Se = standard error, Md = median, IQR = interquartile range.

**Table 3:** Weighted ANCOVA table for COPD severity and MVPA controlling for age, body mass index, sex, accelerometer wearing time, season, work, levels of cotinine, education, income, and ∆ (FEV_indv_−0.7).

	Estimate	SE	95% CI	*t*	*p*
*COPD stages*					
Stage I	0.02	0.09	−0.15–0.21	0.31	0.76
Stage II	−0.3	0.1	−0.59–0.07	−2.49	0.01
Stage ≥ III	−0.8	0.2	−1.31–0.38	−3.52	0.0005
Age	−0.01	0.002	−0.01–0.008	−6.98	0.0001^e−7^
BMI	−0.04	0.005	−0.05–0.03	−7.94	0.0001^e−10^
Sex (women)	−0.3	0.04	−0.34–0.18	−6.26	0.0009^e−6^
Accelerometer wearing	−0.1	0.01	0.07–0.13	6.98	0.0001^e−7^
*Worked last year*					
Study/retired	−0.1	0.2	−0.48–0.2	−0.82	0.41
Working	0.08	0.08	−0.07–0.23	1.1	0.27
*Seasons*					
Spring	−0.03	0.08	−0.18–0.13	−0.32	0.75
Summer	−0.04	0.07	−0.17–0.08	−0.68	0.5
Winter	−0.1	0.1	−0.37–0.08	−1.25	0.21
Marital status (couple)	−0.2	0.06	−0.31–0.05	−2.8	0.005
Levels of cotinine	−0.002	0.0004E−1	−0.0003–0.0001	−4.74	0.0003^e−2^
*Education*					
High school	0.1	0.1	−0.1–0.3	1	0.32
Trade school	0.1	0.1	−0.1–0.3	1.02	0.49
College	0.2	0.1	−0.05–0.38	1.49	0.14
University < bachelor	0.2	0.1	0.05–0.43	2.53	0.01
Bachelor	0.4	0.1	0.16–0.64	3.23	0.001
University > bachelor	0.4	0.1	0.15–0.58	3.35	0.0009
Missing	0.1	0.09	−0.07–0.3	1.17	0.24
*Household income*					
$15k–$19.99k	−0.2	0.2	−0.54–0.23	−0.79	0.43
$20k–$29.99k	−0.2	0.2	−0.48–0.15	−1.01	0.32
$30k–$39.99k	−0.1	0.1	−0.41–0.12	−1.06	0.29
$40k–$49.99k	−0.1	0.1	−0.44–0.15	−0.95	0.34
$50k–$59.99k	−0.2	0.2	−0.5–0.16	−1.02	0.31
$60k–$79.99k	−0.02	0.1	−0.31–0.26	−0.16	0.87
$80k–$99.99k	0.1	0.1	−0.12–0.39	1.02	0.31
≥$100k	0.08	0.1	−0.21–0.37	0.56	0.58
∆ (FEV_indv_−0.7)	−0.4	0.4	−1.17–0.29	−1.18	0.24

## Data Availability

Data access is available only in Statistics Canada Research Data Centers for researchers or students on request (no fees are due). Researchers and students need to run all analysis on-site, and only results approved by the Research Data Center coordinator can be kept by the researchers or students. The application process for data access is available at https://www.statcan.gc.ca/eng/rdc/process.

## Supplementary Materials

Supplementary Materials Supplementary file 1: sample characteristics and detailed results from multivariate analyses. Supplementary file 2: sensitivity analyses with the “healthy control group.” Supplementary file 3: sensitivity analyses with COPD characterized with LLN.
