# Supplementary material for: Daily Objective Physical Activity and Sedentary Time in Adults with COPD Using Spirometry Data from Canadian Measures Health Survey
Source: Can Respir J. 2018 Dec 2;2018:9107435. doi: 10.1155/2018/9107435 (PMC6304807; doi:10.1155/2018/9107435)
Supplement: Supplementary Materials — Supplementary file 1: sample characteristics and detailed results from multivariate analyses. Supplementary file 2: sensitivity analyses with the “healthy control group.” Supplementary file 3: sensitivity analyses with COPD characterized with LLN. [file 9107435.f1.zip › 9107435.f1/Supplementary file 1GHM.docx]

Supplementary file 1

[Descriptive data 2](#__RefHeading___Toc30918_3498084638)

[Weighted sample characteristics for adults with airflow obstruction consistent with COPD GOLD stages and the comparaison group 2](#__RefHeading___Toc31024_3498084638)

[Multivariate analyses for adults with airflow obstruction consistent with COPD 4](#__RefHeading___Toc30920_3498084638)

[Table 1 Weighted ANCOVA table for COPD and MVPA 4](#__RefHeading___Toc31026_3498084638)

[Table 2 Weighted ANCOVA table for COPD and LPA 5](#__RefHeading___Toc31028_3498084638)

[Table 3 Weighted ANCOVA table for COPD and steps 6](#__RefHeading___Toc31030_3498084638)

[Table 4 Weighted ANCOVA table for COPD and sedentary behavior 7](#__RefHeading___Toc31032_3498084638)

[Multivariate analyses for adults with airflow obstruction consistent with 8](#__RefHeading___Toc30922_3498084638)

[COPD GOLD stages 8](#__RefHeading___Toc30924_3498084638)

[Table 5 Weighted ANCOVA table for COPD severity and MVPA 8](#__RefHeading___Toc31034_3498084638)

[Table 6 Weighted ANCOVA table for COPD severity and LPA 9](#__RefHeading___Toc31036_3498084638)

[Table 7 Weighted ANCOVA table for COPD severity and daily steps 10](#__RefHeading___Toc31038_3498084638)

[Table 8 Weighted ANCOVA table for COPD severity and sedentary behavior 11](#__RefHeading___Toc31040_3498084638)

**Descriptive data**

# Weighted sample characteristics for adults with airflow obstruction consistent with COPD GOLD stages and the comparaison group

|  | **Control** | **Stage I** | **Stage II** | **Stage ≥ III** |
| --- | --- | --- | --- | --- |
| Sex (men) %N | 47.8 (7 209 724) | 68.5 (1 112 262) | 34.5 (294 060) | 23.9 (30 940) |
| Age (years) M(Se) | 52.2 (0.2) | 56.9 (0.7) | 57.2 (0.9) | 59.9 (3.4) |
| BMI M(Se) | 27 (0.2) | 25.7 (0.3) | 26.1 (0.5) | 24.4 (1.1) |
| Worked at job last year %(N)  Yes  No  Study/retired | 73.8 (11 120 963)  23.6 (3 554 449)  2.7 (400 151) | 64.9 (1 054 246)  28 (455 280)  7.1 (115 084) | 50.8 (494 523)  44.5 (433 240)  4.7 (45 927) |  |
| Marital status (alone) %(N) | 23.4 (3 521 001) | 35.6 (579 666) | 32.5 (277 043) | 50 (64 806) |
| Income %(N)  < $15k  $15k-$19,99k  $20k-$29,99k  $30k-$39,99k  $40k-$49,99k  $50k-$59,99k  $60k-$79,99k  $80k-$99,99k  ≥ $100k | 3.3 (503 026)  2.1 (314 984)  7.6 (1 145 997)  9.3 (1 406 009)  10 (1 510 219)  8.3 (1 258 115)  15.5 (2 336 038)  12.1 (1 825 625)  31.6 (4 768 563) | 5.3 (86 780)  4.7 (76 630)  9.4 (152 688)  14.4 (233 270)  8.9 (144 290)  11.8 (191 618)  11.6 (189 100)  6.5 (105 125)  27.4 (444 834) | 6.3 (62 172)  4.7 (45 737)  15.5 (152 489)  13 (127 225)  12.5 (122 386)  6.2 (60 698)  15.3 (149 675)  12.5 (122 912)  14 (137 654) |  |
| Education %(N)  Lower than high school  High school  Work school  College  University < Bachelor  University ≥ Bachelor  Missing | 4.5 (684 534)  8.7 (1 320 078)  17 (2 560 089)  9.6 (1 456 458)  12.3 (1 861 439)  17.2 (2 591 464)  30.7 (4 629 193) | 4.3 (69 079)  13.4 (215 695)  19.5 (313 573)  5.2 (83 901)  8.3 (133 391)  12.8 (206 128)  36.6 (588 841) | 2.8 (26 473)  22.3 (214 338)  10.5 (101 072)  8.2 (79 150)  7 (67 584)  10.3 (98 678)  38.8 (372 705) |  |
| **Self reported symptoms and disease** | | | | |
| Self reported COPD %(N) | 0.3 (48 394) | 4.6 (75 156) | 7.5 (63 829) | 26.2 (33 696) |
| Cough phlegm regularly %(N) | 9.9 (1 493 964) | 17.3 (281 069) | 20.9 (178 115) | 55.8 (72 180) |
| Simple chores make short of breath %(N) | 9.1 (1 374 510) | 11.7 (190 532) | 21.5 (182 897) | 71.6 (92 676) |
| Self-rated health %(N)  Fair/poor  (Very)good/excellent | 11.2 (1 687 499)  88.8 (13 381 077) | 9.7 (157 788)  90.3 (1 466 548) | 26.6 (226 156)  73.4 (625 390) | 37.9 (49 092)  62.1 (80 311) |
| Self-rated health compared to 1 year ago %(N)  Much better/somewhat/  about the same  Somewhat/much worse | 86.6 (13 043 443)  13.4 (2 025 133) | 88.2 (1 432 060)  11.8 (192 276) | 77.2 (657 495)  22.8 (194 051) | 64.4 (83 334)  35.6 (46 069) |
| Self reported mood disorder %(N) | 11 (1 653 201) | 9.4 (152 656) | 16 (156 507) |  |
| Other physical or mental condition %(N) | 20.7 (3 123 929) | 15.1 (245 996) | 20.6 (201 959) |  |
| **Psychosocial outcomes** | | | | |
| Self-rated quality of life %(N)  Fair/poor  (Very)good/excellent | 6.1 (915 796)  93.9 (14 158 701) | 6.9 (111 460)  93.1 (1 513 815) | 6.6 (63 886)  93.4 (910 203) |  |
| Self-rated mental health %(N)  Fair/poor  (Very)good/excellent | 5.4 (818 961)  94.6 (14 256 256) | 6.4 (104 230)  93.6 (1 520 595) | 9.4 (91 364)  90.6 (882 454) |  |
| Self-rated stress %(N)  Not at all/not very/ a bit stressful  Quite a bit/extremely stressful | 78.4 (11 813 678)  21.6 (3 249 461) | 76.4 (1 243 051)  23.6 (384 443) | 81.5 (800 848)  18.5 (182 380) |  |
| **Sleep outcomes** |  |  |  |  |
| Sleep duration M(Se) | 7 (0.03) | 7 (0.1) | 7.2 (0.1) | 7.5 (0.3) |
| Frequency of sleep problems  %(N)  Never/rarely/sometimes  Most of the/all the time | 77.8 (11 712 338)  22.2 (3 347 745) | 72.2 (1 176 039)  27.2 (453 593) | 77.6 (764 162)  22.4 (219 986) |  |
| Restorative sleep %(N)  Never/rarely/sometimes  Most of the/all the time | 40.7 (6 127 211)  59.3 (8 932 682) | 62.5 (1 017 990)  37.5 (611 759) | 62.4 (613 883)  37.6 (370 336) |  |
| Difficulty staying awake %(N)  Never/rarely/sometimes  Most of the/all the time | 95 (14 316 265)  5 (746 161) | 95.7 (1 557 908)  543 (70 263) | 93.1 (915 305)  6.9 (67 960) |  |
| **Smoking variables** | | | | |
| Smoking %(N) | 16.1 (2 428 599) | 37.8 (613 085) | 49.5 (422 997) | 66.1 (85 793) |
| Age smoke first whole cig M(Se) | 16 (0.2) | 15.8 (0.3) | 15 (0.3) | 15.3 (0.9) |
| Age smoking everyday M(Se) | 18.9 (0.2) | 19.5 (1) | 17.9 (0.7) | 18.1 (1.3) |
| Number of cig smoked/day when at least one cig/month M(Se) | 6.1 (0.2) | 6.6 (0.4) | 6.7 (0.9) | 9.1 (2.9) |
| Number of years smoked daily  (former daily smokers) M(Se) | 18.3 (0.5) | 21.2 (1.3) | 29.3 (1.2) | 35 (3.5) |
| Levels of cotinine M(Se) | 215.2 (19.1) | 568.6 (73.1) | 653.1 (78.7) | 845.6 (216.4) |
| **Spirometry** | | | | |
| FEV_1_/FVC M(Se) | 0.78 (0.001) | 0.66 (0.002) | 0.62 (0.004) | 0.52 (0.03) |
| FEV_1_ predicted M(Se) | 103.5 (0.4) | 96.5 (0.7) | 69.2 (0.5) | 42.5 (2.2) |
| **Characteristics of physical activity and sedentary** | | | | |
| Acc wearing time (min/day)  M(Se) | 13.7 (0.04) | 13.9 (0.2) | 13.5 (0.1) | 13.5 (0.4) |
| MVPA (min/day) M(Se) | 18.7 (0.6) | 19.6 (1.8) | 11.7 (1.5) | 6.7 (1.5) |
| LPA (min/day) M(Se) | 213.1 (2.8) | 218.6 (6.3) | 215.2 (9.4) | 172.9 (22.6) |
| Steps (steps/day) M(Se) | 7621.2 (111.9) | 8228.1 (294.5) | 6953.2 (335.4) | 6240.2 (876.5) |
| Sed (min/day) M(Se) | 557.3 (2.4) | 560.4 (8.9) | 556.1 (8.8) | 592.2 (28.4) |

**Multivariate analyses for adults with airflow obstruction consistent with COPD**

# **Table 1 Weighted ANCOVA table for COPD and MVPA**

|  | **Estimate** | **SE** | **95% CI** | **t** | ***p*** |
| --- | --- | --- | --- | --- | --- |
| COPD | -0.05 | 0.09 | -0.23 – 0.13 | -0.52 | 0.61 |
| Age | -0.01 | 0.002 | -0.01 – -0.008 | -6.62 | 0.0001E-6 |
| BMI | -0.04 | 0.005 | -0.05 – -0.03 | -8.04 | 0.0007E-11 |
| Sex (women) | -0.3 | 0.04 | -0.4 – -0.19 | -6.73 | 0.0005E-7 |
| Accelerometer wearing | 0.1 | 0.01 | 0.07 – 0.13 | 7.35 | 0.0009E-9 |
| Worked last year  Study/retired  Working | -0.1  0.09 | 0.2  0.08 | -0.47 – 0.21  -0.06 – 0.24 | -0.77  1.2 | 0.44  0.23 |
| Seasons  Spring  Summer  Winter | -0.02  -0.04  -0.1 | 0.08  0.07  0.1 | -0.18 – 0.13  -0.18 – 0.09  -0.37 – 0.09 | -0.31  -0.64  -1.17 | 0.76  0.52  0.24 |
| Marital status (couple) | -0.2 | 0.06 | -0.31 – -0.05 | -2.78 | 0.006 |
| Levels of cotinine | -0.0002 | 0.0004E-1 | -0.0003 – -0.0001 | -4.72 | 0.0003E-2 |
| Education  High school  Work school  College  University < Bachelor  Bachelor  University > Bachelor  Missing | 0.08  0.1  0.2  0.2  0.4  0.4  0.1 | 0.1  0.1  0.1  0.1  0.1  0.1  0.1 | -0.11 – 0.29  -0.09 – 0.3  -0.06 – 0.38  0.05 – 0.43  0.15 – 0.64  0.15 – 0.57  -0.08 – 0.29 | 0.86  1.04  1.44  2.44  3.14  3.34  1.12 | 0.39  0.3  0.15  0.02  0.002  0.001  0.26 |
| Household income  $15k-$19,99k  $20k-$29,99k  $30k-$39,99k  $40k-$49,99k  $50k-$59,99k  $60k-$79,99k  $80k-$99,99k  ≥ $100k | -0.1  -0.2  -0.1  -0.1  -0.2  -0.02  0.1  0.01 | 0.2  0.2  0.1  0.1  0.2  0.1  0.1  0.1 | -0.52 – 0.24  -0.47 – 0.16  -0.4 – 0.13  -0.4 – 0.16  -0.5 – 0.16  -0.3 – 0.27  -0.1 – 0.39  -0.19 – 0.38 | -0.74  -0.96  -0.98  -0.88  -0.97  -0.11  1.04  0.66 | 0.46  0.34  0.33  0.38  0.33  0.91  0.3  0.51 |
| ∆ (FEV_indv_-0.7) | -0.09 | 0.3 | -0.72 – 0.53 | -0.3 | 0.77 |

# **Table 2 Weighted ANCOVA table for COPD and LPA**

|  | **Estimate** | **SE** | **95% CI** | **t** | ***p*** |
| --- | --- | --- | --- | --- | --- |
| COPD | 2.4 | 5.5 | -8.4 – 13.21 | 0.44 | 0.66 |
| Age | -0.8 | 0.2 | -1.05 – -0.45 | -4.92 | 0.0001E-2 |
| BMI | -0.3 | 0.3 | -0.85 – 0.34 | -0.84 | 0.4 |
| Sex (women) | -7.1 | 3.4 | -13.78 – -0.38 | -2.07 | 0.04 |
| Accelerometer wearing | 26.4 | 1.1 | 24.33 – 28.55 | 24.55 | 0.0002E-12 |
| Worked last year  Study/retired  Working | -7.9  17 | 6.4  4 | -20.41 – 4.62  9.06 – 24.88 | -1.24  4.2 | 0.22  0.0003E-1 |
| Seasons  Spring  Summer  Winter | 1.1  1.4  -18.2 | 3.5  6.2  5 | -5.67 – 7.89  -10.81 – 13.6  -27.98 – -8.37 | 0.32  0.22  -3.63 | 0.75  0.82  0.0003 |
| Marital status (couple) | 7.4 | 3.8 | -0.04 – 14.78 | 1.95 | 0.05 |
| Levels of cotinine | -0.006 | 0.003 | -0.01 – 0.0003E-1 | -1.95 | 0.05 |
| Education  High school  Work school  College  University < Bachelor  Bachelor  University > Bachelor  Missing | 19.3  2.9  2.8  -2.8  -8.4  -19  19.7 | 11.4  1  10  9  10.1  11.2  8.2 | -3.06 – 41.75  -17.43 – 23.13  -16.75 – 22.43  -20.4 – 14.8  -28.2 – 11.48  -40.99 – 2.98  3.64 – 35.81 | 1.69  0.28  0.28  -0.31  -0.83  -1.69  2.4 | 0.09  0.78  0.78  0.76  0.41  0.09  0.02 |
| Household income  $15k-$19,99k  $20k-$29,99k  $30k-$39,99k  $40k-$49,99k  $50k-$59,99k  $60k-$79,99k  $80k-$99,99k  ≥ $100k | 0.2  4.6  8.2  12.5  18.4  18.5  19.5  7.4 | 12.1  9.7  8.5  11.7  9.1  8.3  8.6  7.6 | -23.61 – 24  -14.35 – 23.56  -8.39 – 24.82  -10.42 – 35.48  0.5 – 36.35  2.28 – 34.76  2.75 – 36.3  -7.55 – 22.35 | 0.02  0.48  0.97  1.07  2.01  2.24  2.28  0.97 | 0.99  0.63  0.33  0.29  0.04  0.03  0.02  0.33 |
| ∆ (FEV_indv_-0.7) | -20.8 | 25.2 | -70.16 – 28.49 | -0.83 | 0.41 |

# **Table 3 Weighted ANCOVA table for COPD and steps**

|  | **Estimate** | **SE** | **95% CI** | **t** | ***p*** |
| --- | --- | --- | --- | --- | --- |
| COPD | 126.5 | 289.8 | -441.5 – 694.54 | 0.44 | 0.66 |
| Age | -22.9 | 6.1 | -34.82 – -10.92 | -3.75 | 0.0002 |
| BMI | -88.88 | 15.2 | -118.74 – -59.02 | -5.83 | 0.0001E-4 |
| Sex (women) | -998.3 | 144.3 | -1281.12 – -715.44 | -6.92 | 0.0002E-7 |
| Accelerometer wearing | 877.4 | 59 | 761.67 – 993.11 | 14.86 | 0.0002E-12 |
| Worked last year  Study/retired  Working | -99.3  725.9 | 321.7  223.5 | -729.9 – 531.3  287.78 – 1164.03 | -0.31  3.25 | 0.76  0.001 |
| Seasons  Spring  Summer  Winter | 104.6  141  -822.8 | 273  237.3  233.5 | -430.51 – 639.79  -324.03 – 606.09  -1280.36 – -365.24 | 0.38  0.59  -3.52 | 0.7  0.55  0.0005 |
| Marital status (couple) | -232.7 | 202 | -628.6 – 163.13 | -1.15 | 0.25 |
| Levels of cotinine | -0.6 | 0.2 | -0.94 – -0.31 | -3.91 | 0.0001 |
| Education  Highs chool  Work school  College  University < Bachelor  Bachelor  University > Bachelor  Missing | 881.1  447.6  156.2  708.8  722.6  775.9  1080.2 | 402.8  397.2  368.9  340.9  419.3  459  367.6 | 91.75 – 1670.51  -330.93 – 1226.11  -566.76 – 879.23  40.64 – 1376.97  -99.2 – 1544.48  -123.79 – 1675.65  359.69 – 1800.67 | 2.19  1.13  0.42  2.08  1.72  1.69  2.94 | 0.03  0.26  0.67  0.04  0.09  0.09  0.003 |
| Household income  $15k-$19,99k  $20k-$29,99k  $30k-$39,99k  $40k-$49,99k  $50k-$59,99k  $60k-$79,99k  $80k-$99,99k  ≥ $100k | -700.9  -234.4  -212.9  -236.9  125.5  81.7  394.1  325.8 | 563.3  561.3  510.1  604.9  539.8  545.2  459.6  507.1 | -1804.83 – 403.11  -1334.41 – 865.66  -1212.73 – 787.01  -1422.57 – 948.72  -932.51 – 1183.56  -986.77 – 1150.25  -506.7 – 1294.83  -668.17 – 1319.82 | -1.24  -0.42  -0.42  -0.39  0.23  0.15  0.86  0.64 | 0.21  0.68  0.68  0.7  0.82  0.88  0.39  0.52 |
| ∆ (FEV_indv_-0.7) | -850.6 | 1113.8 | -3033.65 – 1332.47 | -0.76 | 0.45 |

# **Table 4 Weighted ANCOVA table for COPD and sedentary behavior**

|  | **Estimate** | **SE** | **95% CI** | **t** | ***p*** |
| --- | --- | --- | --- | --- | --- |
| COPD | -3.7 | 7.7 | -18.78 – 11.46 | -0.48 | 0.64 |
| Age | 1.9 | 0.2 | 1.55 – 2.22 | 11.08 | 0.0002E-12 |
| BMI | 0.4 | 0.3 | -0.27 – 1.08 | 1.18 | 0.24 |
| Sex (women) | 18.2 | 4.3 | 9.81 – 26.51 | 4.26 | 0.0002E-1 |
| Accelerometer wearing | 34.5 | 1.3 | 31.92 – 37.11 | 26.04 | 0.0002E-12 |
| Worked last year  Study/retired  Working | -5.4  -24.6 | 6.5  5.5 | -18.14 – 7.35  -35.31 – -13.89 | -0.83  -4.5 | 0.41  0.0009E-2 |
| Seasons  Spring  Summer  Winter | 0.5  -10.6  12.6 | 5.1  6.7  6.5 | -9.42 – 10.49  -23.79 – 2.62  -0.05 – 25.26 | 0.11  -1.57  1.95 | 0.92  0.12  0.05 |
| Marital status (couple) | 2.3 | 4.5 | -6.54 – 11.19 | 0.51 | 0.61 |
| Levels of cotinine | 0.003 | 0.004 | -0.005 – 0.01 | 0.79 | 0.43 |
| Education  High school  Work school  College  University < Bachelor  Bachelor  University > Bachelor  Missing | -18.5  7.1  6.1  17.3  12.9  12.8  -13.9 | 13  10.3  11.6  8.8  9.8  15  7.8 | -43.92 – 6.91  -13.09 – 27.28  -16.59 – 28.84  0.006 – 34.56  -6.4 – 32.17  -16.6 – 42.24  -29.22 – 1.42 | -1.43  0.69  0.53  1.96  1.31  0.85  -1.78 | 0.15  0.49  0.6  0.05  0.19  0.39  0.08 |
| Household income  $15k-$19,99k  $20k-$29,99k  $30k-$39,99k  $40k-$49,99k  $50k-$59,99k  $60k-$79,99k  $80k-$99,99k  ≥ $100k | 9.9  5.8  -0.7  -6  3.9  -14  -9  -3.2 | 13.4  12.9  12.4  13  12.6  12.2  12.4  11 | -16.35 – 36.14  -19.53 – 31.06  -25.01 – 23.54  -31.4 – 19.39  -20.88 – 28.59  -37.83 – 9.84  -33.33 – 15.23  -24.85 – 18.44 | 0.74  0.45  -0.06  -0.46  0.31  -1.15  -0.73  -0.29 | 0.46  0.66  0.95  0.64  0.76  0.25  0.47  0.77 |
| ∆ (FEV_indv_-0.7) | 13.3 | 30.1 | -45.65 – 72.3 | 0.44 | 0.66 |

**Multivariate analyses for adults with airflow obstruction consistent with**

**COPD GOLD stages**

# **Table 5 Weighted ANCOVA table for COPD severity and MVPA**

|  | **Estimate** | **SE** | **95% CI** | **t** | ***p*** |
| --- | --- | --- | --- | --- | --- |
| COPD stages  Stage I  Stage II  Stage ≥ III | 0.02  -0.3  -0.8 | 0.09  0.1  0.2 | -0.15 – 0.21  -0.59 – -0.07  -1.31 – -0.38 | 0.31  -2.49  -3.52 | 0.76  0.01  0.0005 |
| Age | -0.01 | 0.002 | -0.01 – -0.008 | -6.98 | 0.0001E-7 |
| BMI | -0.04 | 0.005 | -0.05 – -0.03 | -7.94 | 0.0001E-10 |
| Sex (women) | -0.3 | 0.04 | -0.34 – -0.18 | -6.26 | 0.0009E-6 |
| Accelerometer wearing | -0.1 | 0.01 | 0.07 – 0.13 | 6.98 | 0.0001E-7 |
| Worked last year  Study/retired  Working | -0.1  0.08 | 0.2  0.08 | -0.48 – 0.2  -0.07 – 0.23 | -0.82  1.1 | 0.41  0.27 |
| Seasons  Spring  Summer  Winter | -0.03  -0.04  -0.1 | 0.08  0.07  0.1 | -0.18 – 0.13  -0.17 – 0.08  -0.37 – 0.08 | -0.32  -0.68  -1.25 | 0.75  0.5  0.21 |
| Marital status (couple) | -0.2 | 0.06 | -0.31 – -0.05 | -2.8 | 0.005 |
| Levels of cotinine | -0.002 | 0.0004E-1 | -0.0003 – -0.0001 | -4.74 | 0.0003E-2 |
| Education  High school  Work school  College  University < Bachelor  Bachelor  University > Bachelor  Missing | 0.1  0.1  0.2  0.2  0.4  0.4  0.1 | 0.1  0.1  0.1  0.1  0.1  0.1  0.09 | -0.1 – 0.3  -0.1 – 0.3  -0.05 – 0.38  0.05 – 0.43  0.16 – 0.64  0.15 – 0.58  -0.07 – 0.3 | 1  1.02  1.49  2.53  3.23  3.35  1.17 | 0.32  0.49  0.14  0.01  0.001  0.0009  0.24 |
| Household income  $15k-$19,99k  $20k-$29,99k  $30k-$39,99k  $40k-$49,99k  $50k-$59,99k  $60k-$79,99k  $80k-$99,99k  ≥ $100k | -0.2  -0.2  -0.1  -0.1  -0.2  -0.02  0.1  0.08 | 0.2  0.2  0.1  0.1  0.2  0.1  0.1  0.1 | -0.54 – 0.23  -0.48 – 0.15  -0.41 – 0.12  -0.44 – 0.15  -0.5 – 0.16  -0.31 – 0.26  -0.12 – 0.39  -0.21 – 0.37 | -0.79  -1.01  -1.06  -0.95  -1.02  -0.16  1.02  0.56 | 0.43  0.32  0.29  0.34  0.31  0.87  0.31  0.58 |
| ∆ (FEV_indv_-0.7) | -0.4 | 0.4 | -1.17 – 0.29 | -1.18 | 0.24 |

# **Table 6 Weighted ANCOVA table for COPD severity and LPA**

|  | **Estimate** | **SE** | **95% CI** | **t** | ***p*** |
| --- | --- | --- | --- | --- | --- |
| COPD severity stages  Stage I  Stage II  Stage ≥ III | 0.5  6.2  -0.4 | 5.8  9.2  2.2 | -10.87 – 11.9  -11.74 – 24.18  -79.56 – 7.8 | 0.09  0.68  -1.61 | 0.93  0.5  0.11 |
| Age | -0.8 | 0.2 | -1.06 – -0.46 | -5.03 | 0.0007E-3 |
| BMI | -2.6 | 0.3 | -0.84 – -0.33 | -0.86 | 0.39 |
| Sex (women) | -7.1 | 3.5 | -13.83 – -31.44 | -2.05 | 0.04 |
| Accelerometer wearing | 2.6 | 1.1 | 24.32 – 28.57 | 24.42 | 0.0002E-12 |
| Worked last year  Study/retired  Working | -7.3  17 | 6.5  4 | -20.03 – 5.38  9.07 – 24.89 | -1.13  4.21 | 0.26  0.0003E-1 |
| Seasons  Spring  Summer  Winter | 1.06  1.52  -18.18 | 3.45  6.28  4.98 | -5.7 – 7.81  -10.79 – 13.82  -27.95 – -8.42 | 0.31  0.24  -3.65 | 0.76  0.81  0.0003 |
| Marital status (couple) | 7.1 | 3.8 | -0.37 – 14.64 | 1.86 | 0.06 |
| Levels of cotinine | -0.006 | 0.003 | -0.01 – -0.0001 | -1.99 | 0.05 |
| Education  High school  Work school  College  University < Bachelor  Bachelor  University > Bachelor  Missing | 19.2  3.2  3.1  -2.6  -8.3  -18.9  19.8 | 11.3  10.3  9.9  8.9  10.1  11.2  8.2 | -2.92 – 41.23  -16.91 – 23.33  -16.36 – 22.57  -19.96 – 14.86  -28.07 – 11.44  -40.72 – 2.97  3.76 – 35.82 | 1.7  0.31  0.31  -0.29  -0.83  -1.69  2.42 | 0.09  0.75  0.75  0.77  0.41  0.09  0.02 |
| Household income  $15k-$19,99k  $20k-$29,99k  $30k-$39,99k  $40k-$49,99k  $50k-$59,99k  $60k-$79,99k  $80k-$99,99k  ≥ $100k | -0.7  4  7.4  11.9  17.9  17.6  18.8  6.6 | 12  9.5  8.2  11.5  8.8  8  8  7.2 | -24.18 – 22.7  -14.64 – 22.6  -8.68 – 23.46  -10.55 – 34.42  0.61 – 35.22  1.92 – 33.18  3.05 – 34.46  -7.46 – 20.72 | -0.06  0.42  0.9  1.04  2.03  2.2  2.34  0.92 | 0.95  0.68  0.37  0.3  0.04  0.03  0.02  0.36 |
| ∆ (FEV_indv_-0.7) | -31.9 | 27.4 | -85.59 – 21.88 | -1.16 | 0.25 |

# **Table 7 Weighted ANCOVA table for COPD severity and daily steps**

|  | **Estimate** | **SE** | **95% CI** | **t** | ***p*** |
| --- | --- | --- | --- | --- | --- |
| COPD severity stages  Stage I  Stage II  Stage ≥ III | 282.9  -289.8  -765.4 | 305.2  406.9  1048.4 | -315.18 – 881.06  -1087.3 – 507.7  -2820.29 – 1289.5 | 0.93  -0.71  -0.73 | 0.35  0.48  0.47 |
| Age | -23.5 | 6.1 | -35.39 – -11.59 | -3.87 | 0.0001 |
| BMI | -88.1 | 15.2 | -117.95 – -58.35 | -5.8 | 0.0001E-4 |
| Sex (women) | -964.2 | 145.7 | -1249.8 – -678.6 | -6.62 | 0.0001E-6 |
| Accelerometer wearing | 874.4 | 59.9 | 757.03 – 991.82 | 14.6 | 0.0002E-12 |
| Worked last year  Study/retired  Working | -115.4  715.7 | 318.5  225.2 | -739.63 – 508.78  274.28 – 1157.03 | -0.36  3.18 | 0.72  0.002 |
| Seasons  Spring  Summer  Winter | 105.1  135.6  -837.9 | 271.1  238.2  232.8 | -426.28 – 636.5  -331.31 – 602.43  -1294.1 – -381.67 | 0.39  0.57  -3.6 | 0.7  0.57  0.0003 |
| Marital status (couple) | -231.9 | 202.7 | -629.2 – 165.31 | -1.14 | 0.25 |
| Levels of cotinine | -0.6 | 0.2 | -0.94 – -0.31 | -3.86 | 0.0001 |
| Education  High school  Work school  College  University < Bachelor  Bachelor  University > Bachelor  Missing | 906.7  448.1  170  719  735.2  785.4  1089.4 | 400.9  398.3  369  338.2  417.3  459.1  366 | 120.95 – 1692.38  -332.46 – 1228.73  -553.16 – 893.21  56.1 – 1381.9  -82.58 – 1553.07  -114.37 – 1685.16  372.12 – 1806.68 | 2.26  1.13  0.46  2.13  1.76  1.71  2.98 | 0.02  0.26  0.65  0.03  0.08  0.09  0.003 |
| Household income  $15k-$19,99k  $20k-$29,99k  $30k-$39,99k  $40k-$49,99k  $50k-$59,99k  $60k-$79,99k  $80k-$99,99k  ≥ $100k | -708.8  -226.4  -219  -237.2  113.3  87.1  404.5  318.2 | 559.4  558.5  506.8  599.3  537.8  543.9  445.8  499.4 | -1805.06 – 387.56  -1321.05 – 868.17  -1212.36 – 774.45  -1411.88 – 937.41  -940.88 – 1167.44  -978.93 – 1153.05  -469.15 – 1278.16  -660.54 – 1296.96 | -1.27  -0.41  -0.43  -0.4  0.21  0.16  0.91  0.64 | 0.21  0.69  0.67  0.69  0.83  0.87  0.36  0.52 |
| ∆ (FEV_indv_-0.7) | -1428.6 | 1331.7 | -4038.72 – 1181.47 | -1.07 | 0.28 |

# **Table 8 Weighted ANCOVA table for COPD severity and sedentary behavior**

|  | **Estimate** | **SE** | **95% CI** | **t** | ***p*** |
| --- | --- | --- | --- | --- | --- |
| COPD severity stages  Stage I  Stage II  Stage ≥ III | -3.8  -2.2  40.9 | 8.1  10.2  32.5 | -19.73 – 12.14  -22.22 – 17.75  -22.92 – 104.62 | -0.47  -0.22  1.26 | 0.64  0.83  0.21 |
| Age | 1.9 | 0.2 | 1.56 – 2.23 | 11.07 | 0.0002E-12 |
| BMI | 0.4 | 0.3 | -0.27 – 1.07 | 1.17 | 0.24 |
| Sex (women) | 17.8 | 4.3 | 9.26 – 26.25 | 4.1 | 0.0005E-1 |
| Accelerometer wearing | 34.5 | 1.3 | 31.95 – 37.15 | 26.02 | 0.0002E-12 |
| Worked last year  Study/retired  Working | -5.7  -24.5 | 6.5  5.5 | -18.36 – 6.94  -35.2 – -13.78 | -0.89  -4.48 | 0.38  0.0009E-2 |
| Seasons  Spring  Summer  Winter | 0.6  -10.6  12.8 | 5.1  6.8  6.5 | -9.43 – 10.6  -23.94 – 2.68  0.09 – 25.48 | 0.11  -1.57  1.97 | 0.91  0.12  0.05 |
| Marital status (couple) | 2.5 | 4.6 | -6.41 – 11.46 | 0.55 | 0.58 |
| Levels of cotinine | 0.003 | 0.004 | -0.005 – 0.01 | 0.8 | 0.42 |
| Education  High school  Work school  College  University < Bachelor  Bachelor  University > Bachelor  Missing | -18.6  6.8  5.7  16.9  12.7  12.6  -14.1 | 12.7  10.2  11.6  8.8  9.8  15  7.7 | -43.48 – 6.21  -13.33 – 26.85  -16.94 – 28.38  -0.31 – 34.19  -6.47 – 31.83  -16.77 – 41.94  -29.25 – 1.1 | -1.47  0.66  0.5  1.93  1.3  0.84  -1.82 | 0.14  0.51  0.62  0.05  0.19  0.4  0.07 |
| Household income  $15k-$19,99k  $20k-$29,99k  $30k-$39,99k  $40k-$49,99k  $50k-$59,99k  $60k-$79,99k  $80k-$99,99k  ≥ $100k | 10.8  6.2  0.07  -5.5  4.4  -13.2  -8.5  -2.4 | 13.1  12.5  12.1  12.6  12.2  11.8  11.9  10.6 | -14.93 – 36.58  -18.26 – 30.71  -23.66 – 23.81  -30.19 – 19.24  -19.43 – 28.32  -36.27 – 9.88  -31.74 – 14.77  -23.17 – 18.3 | 0.82  0.5  0.006  -0.43  0.37  -1.12  -0.72  -0.23 | 0.41  0.62  1  0.66  0.72  0.26  0.47  0.82 |
| ∆ (FEV_indv_-0.7) | 29.9 | 35.1 | -38.86 – 98.63 | 0.85 | 0.39 |
