# Supplementary material for: Daily Objective Physical Activity and Sedentary Time in Adults with COPD Using Spirometry Data from Canadian Measures Health Survey
Source: Can Respir J. 2018 Dec 2;2018:9107435. doi: 10.1155/2018/9107435 (PMC6304807; doi:10.1155/2018/9107435)
Supplement: Supplementary Materials — Supplementary file 1: sample characteristics and detailed results from multivariate analyses. Supplementary file 2: sensitivity analyses with the “healthy control group.” Supplementary file 3: sensitivity analyses with COPD characterized with LLN. [file 9107435.f1.zip › 9107435.f1/Supplementary file 2GHM.docx]

Supplementary file 2 - Sensivity analyses : Healthy control

[Multivariate analyses for adults with airflow obstruction consistent with COPD 2](#__RefHeading___Toc29644_3498084638)

[Table 1 Weighted ANCOVA table for COPD and MVPA (with a healthy control group) 2](#__RefHeading___Toc29646_3498084638)

[Table 2 Weighted ANCOVA table for COPD and LPA (with a healthy control group) 3](#__RefHeading___Toc29648_3498084638)

[Table 3 Weighted ANCOVA table for COPD and steps (with a healthy control group) 4](#__RefHeading___Toc29650_3498084638)

[Table 4 Weighted ANCOVA table for COPD and sedenatry behavior (with a healthy control group) 5](#__RefHeading___Toc29652_3498084638)

[Multivariate analyses for adults with airflow obstruction consistent with COPD GOLD stages 6](#__RefHeading___Toc29654_3498084638)

[Table 5 Weighted ANCOVA table for COPD severity and MVPA (with a healthy control group) 6](#__RefHeading___Toc29656_3498084638)

[Table 6 Weighted ANCOVA table for COPD severity and LPA (with a healthy control group) 7](#__RefHeading___Toc29658_3498084638)

[Table 7 Weighted ANCOVA table for COPD severity and steps (with a healthy control group) 8](#__RefHeading___Toc29660_3498084638)

[Table 8 Weighted ANCOVA table for COPD severity and sedentary behavior (with a healthy control group) 9](#__RefHeading___Toc29662_3498084638)

[Figure 1 Daily physical activity and sedentary in participants with and without airflow obstruction consistent with COPD . 10](#__RefHeading___Toc29664_3498084638)

[Figure 2 Physical activity (MVPA, LPA) and sedentary levels between control and participants with airflow obstruction consistent with COPD GOLD stages 11](#__RefHeading___Toc29666_3498084638)

[Figure 3 Daily steps and time spent in sedentary behaviors between control and participants with airflow obstruction consistent with COPD GOLD stages 12](#__RefHeading___Toc29668_3498084638)

**Multivariate analyses for adults with airflow obstruction consistent with COPD**

# **Table 1 Weighted ANCOVA table for COPD and MVPA (with a healthy control group)**

|  | **Estimate** | **SE** | **95% CI** | **t** | ***p*** |
| --- | --- | --- | --- | --- | --- |
| COPD | -0.08 | 0.09 | -0.26 – 0.1 | -0.89 | 0.38 |
| Age | -0.01 | 0.002 | -0.01 – -0.007 | -5.61 | 0.0004E-4 |
| BMI | -0.03 | 0.005 | -0.04 – -0.02 | -6.7 | 0.0006E-7 |
| Sex (women) | -0.3 | 0.04 | -0.34 – -0.18 | -6.22 | 0.0001E-5 |
| Accelerometer wearing | 0.1 | 0.01 | 0.06 – 0.12 | 6.87 | 0.0002E-7 |
| Worked last year  Study/retired  Working | 0.3  0.1 | 0.2  0.07 | -0.35 – 0.4  -0.03 – 0.25 | 0.15  1.56 | 0.88  0.12 |
| Seasons  Spring  Summer  Winter | -0.006  -0.08  -0.1 | 0.08  0.07  0.1 | -0.17 – 0.16  -0.22 – 0.06  -0.35 – 0.14 | -0.08  -1.08  -0.84 | 0.94  0.28  0.4 |
| Marital status (couple) | -0.2 | 0.07 | -0.34 – -0.09 | -3.27 | 0.001 |
| Levels of cotinine | -0.0002 | 0.0004E-1 | -0.0002 – -0.00007 | -3.63 | 0.0003 |
| Education  High school  Work school  College  University < Bachelor  Bachelor  University > Bachelor  Missing | 0.08  0.1  0.2  0.3  0.5  0.4  0.2 | 0.1  0.1  0.1  0.1  0.1  0.1  0.1 | -0.05 – 0.38  -0.03 – 0.4  -0.07 – 0.4  0.09 – 0.5  0.17 – 0.74  0.16 – 0.68  -0.02 – 0.39 | 1.51  1.72  1.36  2.8  3.11  3.15  1.74 | 0.13  0.09  0.17  0.005  0.002  0.002  0.08 |
| Household income  $15k-$19,99k  $20k-$29,99k  $30k-$39,99k  $40k-$49,99k  $50k-$59,99k  $60k-$79,99k  $80k-$99,99k  ≥ $100k | -0.1  -0.2  -0.1  -0.1  -0.2  -0.03  0.1  0.1 | 0.2  0.2  0.1  0.2  0.2  0.2  0.2  0.2 | -0.59 – 0.31  -0.56 – 0.16  -0.42 – 0.17  -0.45 – 0.22  -0.53 – 0.16  -0.35 – 0.29  -0.22 – 0.42  -0.22 – 0.41 | -0.61  -1.08  -0.85  -0.65  -1.04  -0.18  0.61  0.6 | 0.54  0.28  0.4  0.52  0.3  0.86  0.54  0.55 |
| ∆ (FEV_indv_-0.7) | -0.07 | 0.4 | -0.77 – 0.64 | -0.19 | 0.85 |

# **Table 2 Weighted ANCOVA table for COPD and LPA (with a healthy control group)**

|  | **Estimate** | **SE** | **95% CI** | **t** | ***p*** |
| --- | --- | --- | --- | --- | --- |
| COPD | 0.25 | 5.9 | -11.32 – 11.82 | 0.04 | 0.97 |
| Age | -0.73 | 0.2 | -1.1 – -0.36 | -3.86 | 0.0001 |
| BMI | -0.1 | 0.4 | -0.81 – 0.61 | -0.27 | 0.79 |
| Sex (women) | -6.8 | 3.6 | -13.9 – 0.38 | -1.86 | 0.06 |
| Accelerometer wearing | 26.2 | 1.2 | 23.88 – 28.51 | 22.21 | 0.0002E-12 |
| Worked last year  Study/retired  Working | -10.8  14.9 | 7.8  4.8 | -26.17 – 4.49  5.4 – 24.29 | -1.39  3.08 | 0.17  0.002 |
| Seasons  Spring  Summer  Winter | 1.1  0.22  -19.3 | 4.1  6.8  5.3 | -6.85 – 9.15  -13.17 – 13.61  -29.64 – -8.94 | 0.28  0.03  -3.65 | 0.78  0.97  0.0003 |
| Marital status (couple) | 8.3 | 4.2 | 0.03 – 16.5 | 1.97 | 0.05 |
| Levels of cotinine | -0.005 | 0.003 | -0.01 – 0.002 | -1.32 | 0.19 |
| Education  High school  Work school  College  University < Bachelor  Bachelor  University > Bachelor  Missing | 19.2  2.1  0.5  -1.2  -7.4  -21.3  21.3 | 13.8  12.6  12.7  11.3  12.7  13.1  10.8 | -7.81 – 46.22  -22.64 – 26.79  -24.4 – 25.36  -23.26 – 20.92  -32.33 – 17.53  -46.98 – 4.42  0.14 – 42.36 | 1.39  0.17  0.04  -0.1  -0.58  -1.62  1.97 | 0.16  0.87  0.97  0.92  0.56  0.11  0.05 |
| Household income  $15k-$19,99k  $20k-$29,99k  $30k-$39,99k  $40k-$49,99k  $50k-$59,99k  $60k-$79,99k  $80k-$99,99k  ≥ $100k | 2.1  9  13.9  19.6  22.2  24.8  23.4  13 | 13.4  9.3  9  12  9.3  9  9.1  7.6 | -24.07 – 28.35  -9.19 – 27.13  -3.69 – 31.44  -3.9 – 43.08  3.88 – 40.44  7.11 – 42.42  5.61 – 41.27  -1.77 – 27.85 | 0.16  0.97  1.55  1.64  2.38  2.75  2.58  1.73 | 0.87  0.33  0.12  0.1  0.02  0.006  0.01  0.08 |
| ∆ (FEV_indv_-0.7) | -19.1 | 27.5 | -72.92 – 34.8 | -0.69 | 0.49 |

# **Table 3 Weighted ANCOVA table for COPD and steps (with a healthy control group)**

|  | **Estimate** | **SE** | **95% CI** | **t** | ***p*** |
| --- | --- | --- | --- | --- | --- |
| COPD | -33.8 | 303.3 | -628.3 – 560.78 | -0.11 | 0.91 |
| Age | -19.8 | 7.8 | -35 – -4.56 | -2.55 | 0.01 |
| BMI | -80.1 | 17.8 | -115.02 – -45.17 | -4.5 | 0.0009E-2 |
| Sex (women) | -968.5 | 167 | -1295.84 – -641.2 | -5.8 | 0.0001E-4 |
| Accelerometer wearing | 860.2 | 62.7 | 737.24 – 983.13 | 13.7 | 0.0002E-12 |
| Worked last year  Study/retired  Working | 98.9  711.8 | 411.7  236.7 | -707.96 – 905.71  247.95 – 1175.74 | 0.2  3 | 0.81  0.003 |
| Seasons  Spring  Summer  Winter | 133.3  28.8  -831.8 | 314.2  310.4  265.6 | -482.52 – 749.05  -579.63 – 637.18  -1352.48 – -311.2 | 0.42  0.09  -3.13 | 0.67  0.93  0.002 |
| Marital status (couple) | -361.1 | 235.3 | -822.29 – 100.05 | -1.54 | 0.13 |
| Levels of cotinine | -0.5 | 0.2 | -0.87 – -0.22 | -3.25 | 0.001 |
| Education  High school  Work school  College  University < Bachelor  Bachelor  University > Bachelor  Missing | 968.8  408.6  93.9  733.6  790.6  861.1  1235.4 | 450.2  453.5  463.1  430.3  547  546.6  406.2 | 86.47 – 1851.1  -480.18 – 1297.46  -813.74 – 1001.54  -109.72 – 1576.99  -281.45 – 1862.61  -210.18 – 1932.38  439.19 – 2031.58 | 2.15  0.9  0.2  1.71  1.45  1.58  3.04 | 0.03  0.37  0.84  0.09  0.15  0.12  0.002 |
| Household income  $15k-$19,99k  $20k-$29,99k  $30k-$39,99k  $40k-$49,99k  $50k-$59,99k  $60k-$79,99k  $80k-$99,99k  ≥ $100k | -632  -158.8  -70.9  38.2  287.5  275.5  517.2  564.3 | 697.8  671.5  629.9  736.7  665.5  687.5  649.3  614.2 | -1999.66 – 735.67  -1475.01 – 1157.33  -1305.48 – 1163.67  -1405.7 – 1482.02  -1016.9 – 1591.77  -1072 – 1623.1  -755.37 – 1789.69  -639.53 – 1768.12 | -0.91  -0.24  -0.11  0.05  0.43  0.4  0.8  0.92 | 0.37  0.81  0.91  0.96  0.67  0.69  0.43  0.36 |
| ∆ (FEV_indv_-0.7) | -977.84 | 1315.9 | -3556.88 – 1601.2 | -0.74 | 0.46 |

# **Table 4 Weighted ANCOVA table for COPD and sedenatry behavior (with a healthy control group)**

|  | **Estimate** | **SE** | **95% CI** | **t** | ***p*** |
| --- | --- | --- | --- | --- | --- |
| COPD | -1.7 | 8.3 | -18.01 – 14.54 | -0.21 | 0.83 |
| Age | 1.9 | 0.2 | 1.49 – 2.32 | 9.04 | 0.0002E-12 |
| BMI | 0.3 | 0.4 | -0.61 – 1.14 | 0.59 | 0.56 |
| Sex (women) | 16.6 | 4.7 | 7.45 – 25.68 | 3.56 | 0.0004 |
| Accelerometer wearing | 35 | 1.4 | 32.15 – 37.77 | 24.37 | 0.0002E-12 |
| Worked last year  Study/retired  Working | -11.1  -22.6 | 7.4  5.8 | -25.59 – 3.31  -34 – -11.17 | -1.51  -3.88 | 0.13  0.0001 |
| Seasons  Spring  Summer  Winter | 1.1  -10.9  10.6 | 5.7  7.9  6.2 | -10.1 – 12.23  -26.32 – 4.52  -1.62 – 22.85 | 0.19  -1.39  1.7 | 0.85  0.17  0.09 |
| Marital status (couple) | 3.1 | 5.3 | -7.27 – 13.55 | 0.59 | 0.55 |
| Levels of cotinine | 0.002 | 0.004 | -0.007 – 0.01 | 0.41 | 0.68 |
| Education  Highschool  Workschool  College  University < Bachelor  Bachelor  University > Bachelor  Missing | -25.8  2.5  2.7  10.8  5  11.5  -21.3 | 15  12.1  13.2  10.6  11.4  17.4  9.9 | -55.13 – 3.61  -21.23 – 26.22  -23.23 – 28.7  -9.94 – 31.63  -17.39 – 27.45  -22.68 – 45.66  -40.62 – -1.96 | -1.72  0.21  0.21  1.02  0.44  0.66  -2.16 | 0.09  0.84  0.84  0.31  0.66  0.51  0.03 |
| Household income  $15k-$19,99k  $20k-$29,99k  $30k-$39,99k  $40k-$49,99k  $50k-$59,99k  $60k-$79,99k  $80k-$99,99k  ≥ $100k | 9.6  6.7  -3.4  -9.9  3.9  -17.7  -10.1  -7.3 | 16.2  15.5  14.1  15.6  15.4  14.9  15.8  13.4 | -22.08 – 41.23  -23.75 – 37.2  -31.04 – 24.19  -40.44 – 20.6  -26.34 – 34.08  -46.95 – 11.6  -41.08 – 20.78  -33.57 – 19.01 | 0.59  0.43  -0.24  -0.64  0.25  -1.18  -0.64  -0.54 | 0.55  0.67  0.81  0.52  0.8  0.24  0.52  0.59 |
| ∆ (FEV_indv_-0.7) | 10.5 | 32.1 | -52.31 – 73.41 | 0.33 | 0.74 |

**Multivariate analyses for adults with airflow obstruction consistent with COPD GOLD stages**

# **Table 5 Weighted ANCOVA table for COPD severity and MVPA (with a healthy control group)**

|  | **Estimate** | **SE** | **95% CI** | **t** | ***p*** |
| --- | --- | --- | --- | --- | --- |
| COPD stages  Stage I  Stage II  Stage ≥ III | -0.01  -0.4  -0.9 | 0.09  0.1  0.2 | -0.2 – 0.17  -0.65 – -0.11  -1.39 – -0.41 | -0.11  -2.73  -3.61 | 0.91  0.007  0.0003 |
| Age | -0.01 | 0.002 | -0.01 – -0.007 | -5.93 | 0.0006E-5 |
| BMI | -0.03 | 0.005 | -0.04 – -0.02 | -6.51 | 0.0002E-6 |
| Sex (women) | -0.2 | 0.04 | -0.32 – -0.16 | -5.67 | 0.0002E-4 |
| Accelerometer wearing | 0.9 | 0.01 | 0.06 – 0.12 | 6.5 | 0.0002E-6 |
| Worked last year  Study/retired  Working | 0.02  0.1 | 0.2  0.07 | -0.36 – 0.4  -0.04 – 0.25 | 0.09  1.43 | 0.93  0.15 |
| Seasons  Spring  Summer  Winter | -0.01  -0.1  -0.1 | 0.08  0.07  0.1 | -0.17 – 0.15  -0.22 – 0.06  -0.35 – 0.13 | -0.09  -1.12  -0.92 | 0.93  0.26  0.36 |
| Marital status (couple) | -0.2 | 0.07 | -0.34 – -0.09 | -3.29 | 0.001 |
| Levels of cotinine | -0.0002 | 0.0004E-1 | -0.0002 – -0.00007 | -3.58 | 0.0004 |
| Education  High school  Work school  College  University < Bachelor  Bachelor  University > Bachelor  Missing | 0.2  0.2  0.2  0.3  0.5  0.4  0.2 | 0.1  0.1  0.1  0.1  0.1  0.1  0.1 | -0.03 – 0.39  -0.03 – 0.38  -0.07 – 0.41  0.1 – 0.5  0.18 – 0.74  0.16 – 0.68  -0.02 – 0.39 | 1.65  1.69  1.41  2.92  3.18  3.19  1.79 | 0.1  0.09  0.16  0.004  0.002  0.002  0.07 |
| Household income  $15k-$19,99k  $20k-$29,99k  $30k-$39,99k  $40k-$49,99k  $50k-$59,99k  $60k-$79,99k  $80k-$99,99k  ≥ $100k | -0.2  -0.2  -0.1  -0.1  -0.2  -0.04  0.1  0.08 | 0.2  0.2  0.2  0.2  0.2  0.2  0.2  0.2 | -0.61 – 0.3  -0.57 – 0.15  -0.44 – 0.15  -0.46 – 0.21  -0.55 – 0.15  -0.36 – 0.28  -0.23 – 0.41  -0.24 – 0.39 | -0.68  -1.14  -0.96  -0.75  -1.11  -0.25  0.58  0.48 | 0.5  0.25  0.34  0.46  0.27  0.81  0.57  0.63 |
| ∆ (FEV_indv_-0.7) | -0.5 | 0.4 | -1.32 – 0.39 | -1.07 | 0.29 |

# **Table 6 Weighted ANCOVA table for COPD severity and LPA (with a healthy control group)**

|  | **Estimate** | **SE** | **95% CI** | **t** | ***p*** |
| --- | --- | --- | --- | --- | --- |
| COPD stages  Stage I  Stage II  Stage ≥ III | -1.6  3.1  -37.9 | 28.6  6.3  9.4 | -13.85 – 10.7  -15.29 – 21.45  -83 – 7.25 | -0.25  0.33  -1.65 | 0.08  0.74  0.1 |
| Age | -0.7 | 0.2 | -1.11 – -0.37 | -3.9 | 0.0001 |
| BMI | -0.1 | 0.4 | -0.79 – 0.59 | -0.27 | 0.78 |
| Sex (women) | -6.7 | 3.7 | -13.96 – 0.55 | -1.81 | 0.07 |
| Accelerometer wearing | 26.2 | 1.2 | 23.86 – 28.53 | 22 | 0.0002E-12 |
| Worked last year  Study/retired  Working | -10  14.9 | 8.1  4.9 | -25.83 – 5.82  5.34 – 24.36 | -1.24  3.1 | 0.22  0.002 |
| Seasons  Spring  Summer  Winter | 1.1  0.4  -19.3 | 4.1  6.9  5.3 | -6.91 – 9.08  -13.12 – 13.85  -29.61 – -9.03 | 0.27  0.05  -3.68 | 0.79  0.96  0.0003 |
| Marital status (couple) | 8 | 4.3 | -0.41 – 16.35 | 1.86 | 0.06 |
| Levels of cotinine | -0.005 | 0.003 | -0.01 – 0.002 | -1.35 | 0.18 |
| Education  High school  Work school  College  University < Bachelor  Bachelor  University > Bachelor  Missing | 19  2.5  0.8  -0.9  -7.4  -21.1  21.3 | 13.6  12.5  12.6  11.1  12.6  13  10.7 | -7.55 – 45.6  -21.96 – 26.88  -23.89 – 25.44  -22.64 – 20.85  -32.14 – 17.42  -46.63 – 4.36  0.3 – 42.3 | 1.4  0.2  0.06  -0.08  -0.58  -1.63  1.99 | 0.16  0.84  0.95  0.94  0.56  0.1  0.05 |
| Household income  $15k-$19,99k  $20k-$29,99k  $30k-$39,99k  $40k-$49,99k  $50k-$59,99k  $60k-$79,99k  $80k-$99,99k  ≥ $100k | 0.9  8.1  12.7  18.9  21.4  23.5  22.4  12 | 13.2  9.1  8.6  11.7  8.8  8.6  8.3  6.9 | -24.92 – 26.77  -9.69 – 25.89  -4.06 – 29.5  -4.13 – 41.63  4.05 – 38.72  6.57 – 40.42  6.1 – 38.72  -1.64 – 25.6 | 0.07  0.89  1.49  1.61  2.42  2.72  2.69  1.73 | 0.94  0.37  0.14  0.11  0.02  0.003  0.007  0.09 |
| ∆ (FEV_indv_-0.7) | -32 | 30.5 | -1.32 – 0.39 | -1.05 | 0.29 |

# **Table 7 Weighted ANCOVA table for COPD severity and steps (with a healthy control group)**

|  | **Estimate** | **SE** | **95% CI** | **t** | ***p*** |
| --- | --- | --- | --- | --- | --- |
| COPD stages  Stage I  Stage II  Stage ≥ III | 123.2  -497.7  -1009.1 | 325.2  415.4  1069.7 | -514.22 – 760.58  -1311.78 – 316.46  -3105.59 – 1087.38 | 0.38  -1.2  -0.94 | 0.71  0.23  0.35 |
| Age | -20.5 | 7.8 | -35.78 – -5.32 | -2.64 | 0.008 |
| BMI | -78.8 | 17.7 | -113.57 – -44.08 | -4.45 | 0.0001E-1 |
| Sex (women) | -923.7 | 170 | -1256.91 – -590.53 | -5.43 | 0.0009E-4 |
| Accelerometer wearing | 856.3 | 63.6 | 731.63 – 980.98 | 13.46 | 0.0002E-12 |
| Worked last year  Study/retired  Working | 74.3  696.8 | 411.4  239.5 | -732.06 – 880.67  227.35 – 1166.28 | 0.18  2.91 | 0.86  0.004 |
| Seasons  Spring  Summer  Winter | 133.8  22.4  -851.1 | 311.4  310.3  264.3 | -476.4 – 744.09  -585.84 – 630.63  -1369.13 – -333.06 | 0.43  0.07  -3.22 | 0.67  0.94  0.001 |
| Marital status (couple) | -361.1 | 236.5 | -824.65 – 102.46 | -1.53 | 0.13 |
| Levels of cotinine | -0.5 | 0.17 | -0.88 – -0.21 | -3.18 | 0.002 |
| Education  High school  Work school  College  University < Bachelor  Bachelor  University > Bachelor  Missing | 1000.8  408.6  110.4  745.7  805.6  871.7  1245.5 | 450.4  451.9  460.8  423.2  544.8  546  404 | 118 – 1883.6  -477.14 – 1294.39  -792.66 – 1013.45  -83.74 – 1575.16  -262.14 – 1873.35  -198.46 – 1941.86  453.73 – 2037.21 | 2.22  0.9  0.24  1.76  1.48  1.6  3.08 | 0.03  0.37  0.81  0.08  0.14  0.11  0.002 |
| Household income  $15k-$19,99k  $20k-$29,99k  $30k-$39,99k  $40k-$49,99k  $50k-$59,99k  $60k-$79,99k  $80k-$99,99k  ≥ $100k | -648.7  -149.5  -84  32.3  267.2  278.6  526.9  550.9 | 690.4  669.6  628.2  729.4  663.7  689.4  637.8  606.2 | -2001.75 – 704.4  -1461.89 – 1162.83  -1315.19 – 1147.26  -1397.32 – 1461.91  -1033.54 – 1568.03  -1072.62 – 1629.82  -723.18 – 1777.03  -637.27 – 1739.01 | -0.94  -0.22  -0.13  0.04  0.4  0.4  0.83  0.91 | 0.35  0.82  0.89  0.96  0.69  0.69  0.41  0.36 |
| ∆ (FEV_indv_-0.7) | -1687.4 | 0.4 | -4807.33 – 1432.57 | -1.06 | 0.29 |

# **Table 8 Weighted ANCOVA table for COPD severity and sedentary behavior (with a healthy control group)**

|  | **Estimate** | **SE** | **95% CI** | **t** | ***p*** |
| --- | --- | --- | --- | --- | --- |
| COPD stages  Stage I  Stage II  Stage ≥ III | -1.8  0.7  43.5 | 8.7  10.8  32.7 | -18.81 – 15.29  -20.51 – 21.88  -20.65 – 107.71 | -0.2  0.06  1.33 | 0.84  0.95  0.18 |
| Age | 1.9 | 0.2 | 1.5 – 2.34 | 8.97 | 0.0002E-12 |
| BMI | 0.2 | 0.4 | -0.63 – 1.13 | 0.56 | 0.58 |
| Sex (women) | 16 | 4.8 | 6.67 – 25.4 | 3.36 | 0.0009 |
| Accelerometer wearing | 35 | 1.4 | 32.18 – 37.83 | 24.28 | 0.0002E-12 |
| Worked last year  Study/retired  Working | -11.6  -22.4 | 7.4  5.8 | -26.1 – 2.83  -33.85 – -11.01 | -1.58  -3.85 | 0.12  0.0001 |
| Seasons  Spring  Summer  Winter | 1.1  -11  10.9 | 5.7  7.9  6.3 | -10.15 – 12.38  -26.47 – 4.54  -1.43 – 23.14 | 0.19  -1.39  1.73 | 0.85  0.17  0.08 |
| Marital status (couple) | 3.4 | 5.4 | -7.1 – 13.92 | 0.64 | 0.52 |
| Levels of cotinine | 0.002 | 0.004 | -0.007 – 0.01 | 0.42 | 0.68 |
| Education  High school  Work school  College  University < Bachelor  Bachelor  University > Bachelor  Missing | -25.9  2.1  2.3  10.5  4.8  11.2  -21.5 | 14.6  12  13.2  10.5  11.4  17.4  9.8 | -54.66 – 2.76  -21.45 – 25.73  -23.61 – 28.14  -10.21 – 31.12  -17.48 – 27.12  -22.84 – 45.3  -40.66 – -2.25 | -1.77  0.18  0.17  0.99  0.42  0.65  -2.19 | 0.08  0.86  0.86  0.32  0.67  0.52  0.03 |
| Household income  $15k-$19,99k  $20k-$29,99k  $30k-$39,99k  $40k-$49,99k  $50k-$59,99k  $60k-$79,99k  $80k-$99,99k  ≥ $100k | 10.9  7.4  -2.2  -9.1  4.8  -16.5  -9.3  -6.2 | 15.8  15  13.6  15  14.7  14.4  15  12.7 | -20.02 – 41.76  -21.89 – 36.72  -28.79 – 24.33  -38.51 – 20.33  -24.1 – 33.69  -44.77 – 11.67  -38.77 – 20.13  -31.07 – 18.72 | 0.69  0.5  -0.16  -0.61  0.33  -1.15  -0.62  -0.49 | 0.5  0.62  0.87  0.55  0.75  0.25  0.54  0.63 |
| ∆ (FEV_indv_-0.7) | 30 | 38 | -44.55 – 104.56 | 0.79 | 0.43 |

# Figure 1 Daily physical activity and sedentary in participants with and without airflow obstruction consistent with COPD .

# Figure 2 Physical activity (MVPA, LPA) and sedentary levels between control and participants with airflow obstruction consistent with COPD GOLD stages

# Figure 3 Daily steps and time spent in sedentary behaviors between control and participants with airflow obstruction consistent with COPD GOLD stages
