# Supplementary material for: Daily Objective Physical Activity and Sedentary Time in Adults with COPD Using Spirometry Data from Canadian Measures Health Survey
Source: Can Respir J. 2018 Dec 2;2018:9107435. doi: 10.1155/2018/9107435 (PMC6304807; doi:10.1155/2018/9107435)
Supplement: Supplementary Materials — Supplementary file 1: sample characteristics and detailed results from multivariate analyses. Supplementary file 2: sensitivity analyses with the “healthy control group.” Supplementary file 3: sensitivity analyses with COPD characterized with LLN. [file 9107435.f1.zip › 9107435.f1/Supplementary file 3GHM.docx]

Supplementary file 3

[Descriptive data 2](#__RefHeading___Toc31146_3498084638)

[Weighted ≥ 35 years old characteristics of the Canadian Health Measures Survey (cycle 1,2,3) 2](#__RefHeading___Toc31148_3498084638)

[Multivariate analyses for adults with airflow obstruction consistent with COPD me](#__RefHeading___Toc31159_3498084638)asured with LLN [3](#__RefHeading___Toc31159_3498084638)

[Table 1 Weighted ANCOVA table for COPD mesured with LLN and MVPA 3](#__RefHeading___Toc31150_3498084638)

[Table 2 Weighted ANCOVA table for COPD mesured with LLN and LPA 4](#__RefHeading___Toc31152_3498084638)

[Table 3 Weighted ANCOVA table for COPD mesured with LLN and steps 5](#__RefHeading___Toc31154_3498084638)

[Table 4 Weighted ANCOVA table for COPD mesured with LLN and sedentary behavior 6](#__RefHeading___Toc31156_3498084638)

**Descriptive data**

# Weighted ≥ 35 years old characteristics of the Canadian Health Measures Survey (cycle 1,2,3)

|  | **MVPA (min/day)** | | | | **LPA (min/day)** | | | | | **Steps (steps/day)** | | | | | **Sed (min/day)** | | | |
| --- | --- | --- | --- | --- | --- | --- | --- | --- | --- | --- | --- | --- | --- | --- | --- | --- | --- | --- |
|  | M | Se | Md | IQTR | M | Se | Md | IQTR | M | | Se | Md | IQTR | M | | Se | Md | IQTR |
| **Control** | 18.5 | 0.6 | 13 | 20.8 | 212.3 | 2.6 | 203 | 103.3 | 7590 | | 109.1 | 7095.2 | 4733.7 | 558.4 | | 2.4 | 564.5 | 121.4 |
| **COPD**  **LLN** | 17.4 | 1.9 | 10 | 20.9 | 224 | 6.4 | 201.8 | 126.7 | 8058 | | 353.5 | 7253.5 | 5665 | 552.2 | | 7 | 551.7 | 137.9 |

**Multivariate analyses for adults with airflow obstruction consistent with COPD mesured with LLN**

# **Table 1 Weighted ANCOVA table for COPD mesured with LLN and MVPA**

|  | **Estimate** | **SE** | **95% CI** | **t** | ***p*** |
| --- | --- | --- | --- | --- | --- |
| COPDLLN | -0.01 | 0.11 | -0.24 – 0.21 | -0.11 | 0.91 |
| Age | -0.01 | 0.002 | -0.01 – -0.008 | -6.53 | 0.0002E-6 |
| BMI | -0.04 | 0.005 | -0.05 – -0.03 | -8.06 | 0.0006E-11 |
| Sex (women) | -0.27 | 0.04 | -0.35 – -0.2 | -6.78 | 0.0004E-7 |
| Accelerometer wearing | 0.1 | 0.01 | 0.07 – 0.13 | 7.28 | 0.0001E-8 |
| Worked last year  Study/retired  Working | -0.13  0.09 | 0.17  0.07 | -0.48 – 0.21  -0.06 – 0.24 | -0.77  1.22 | 0.44  0.22 |
| Seasons  Spring  Summer  Winter | -0.03  -0.04  -0.14 | 0.08  0.08  0.07 | -0.18 – 0.13  -0.17 – 0.09  -0.37 – 0.09 | -0.32  -0.62  -1.17 | 0.75  0.53  0.24 |
| Marital status (couple) | -0.18 | 0.06 | -0.31 – -0.05 | -2.78 | 0.006 |
| Levels of cotinine | -0.0001 | 0.0004E-1 | -0.0003 – -0.0001 | -4.67 | 0.0004E-2 |
| Education  High school  Work school  College  University < Bachelor  Bachelor  University > Bachelor  Missing | 0.09  0.1  0.16  0.24  0.4  0.36  0.11 | 0.1  0.1  0.11  0.1  0.13  0.11  0.1 | -0.11 – 0.29  -0.09 – 0.3  -0.06 – 0.38  0.05 – 0.43  0.15 – 0.64  0.15 – 0.57  -0.08 – 0.29 | 0.86  1.03  1.46  2.44  3.15  3.33  1.12 | 0.39  0.3  0.15  0.01  0.002  0.0009  0.26 |
| Household income  $15k-$19,99k  $20k-$29,99k  $30k-$39,99k  $40k-$49,99k  $50k-$59,99k  $60k-$79,99k  $80k-$99,99k  ≥ $100k | -0.14  -0.15  -0.13  -0.13  -0.16  -0.02  0.14  0.1 | 0.19  0.16  0.14  0.15  0.16  0.14  0.13  0.14 | -0.52 – 0.23  -0.47 – 0.16  -0.4 – 0.13  -0.42 – 0.16  -0.48 – 0.16  -0.29 – 0.26  -0.12 – 0.39  -0.19 – 0.38 | -0.75  -0.97  -0.98  -0.89  -1  -0.11  1.05  0.67 | 0.46  0.33  0.33  0.37  0.32  0.91  0.29  0.5 |
| ∆ (FEV_indv_-0.7) | 0.03 | 0.34 | -0.64 – 0.7 | 0.08 | 0.93 |

# **Table 2 Weighted ANCOVA table for COPD mesured with LLN and LPA**

|  | **Estimate** | **SE** | **95% CI** | **t** | ***p*** |
| --- | --- | --- | --- | --- | --- |
| COPDLLN | 7.47 | 6.2 | -4.68 – 19.62 | 1.21 | 0.91 |
| Age | -0.73 | 0.15 | -1.02 – -0.44 | -4.94 | 0.0002E-6 |
| BMI | -0.25 | 0.3 | -0.84 – 0.34 | -0.84 | 0.0006E-11 |
| Sex (women) | -7.21 | 3.42 | -13.92 – -0.51 | -2.11 | 0.0004E-7 |
| Accelerometer wearing | 26.42 | 1.08 | 24.31 – 28.53 | 24.53 | 0.0001E-8 |
| Worked last year  Study/retired  Working | -8.08  17 | 6.36  4.02 | -20.55 – 4.38  9.12 – 24.88 | -1.27  4.23 | 0.44  0.22 |
| Seasons  Spring  Summer  Winter | 1.16  1.45  -18.21 | 3.44  6.21  5.03 | -5.58 – 7.89  -10.72 – 13.63  -28.07 – -8.34 | 0.34  0.23  -3.62 | 0.75  0.53  0.24 |
| Marital status (couple) | 7.28 | 3.8 | -0.16 – 14.73 | 1.92 | 0.006 |
| Levels of cotinine | -0.006 | 0.003 | -0.01 – -0.0004E-2 | -1.96 | 0.0004E-2 |
| Education  High school  Work school  College  University < Bachelor  Bachelor  University > Bachelor  Missing | 19.13  2.88  2.78  -2.79  -8.43  -19.1  19.63 | 11.37  10.33  10.02  8.96  10.11  11.19  8.17 | -3.16 – 41.42  -17.36 – 23.12  -16.86 – 22.41  -20.36 – 14.78  -28.25 – 11.4  -41.02 – 2.83  3.61 – 35.64 | 1.68  0.28  0.28  -0.31  -0.83  -1.71  2.4 | 0.39  0.3  0.15  0.01  0.002  0.0009  0.26 |
| Household income  $15k-$19,99k  $20k-$29,99k  $30k-$39,99k  $40k-$49,99k  $50k-$59,99k  $60k-$79,99k  $80k-$99,99k  ≥ $100k | 0.18  4.56  8.01  12.71  18.68  18.57  19.59  7.51 | 12.13  9.6  8.43  11.61  9.12  8.24  8.53  7.6 | -23.6 – 23.95  -14.25 – 23.37  -8.51 – 24.53  -10.04 – 35.46  0.8 – 36.56  2.42 – 34.71  2.87 – 36.32  -7.39 – 22.41 | 0.02  0.48  0.95  1.1  2.05  2.25  2.3  0.99 | 0.46  0.33  0.33  0.37  0.32  0.91  0.29  0.5 |
| ∆ (FEV_indv_-0.7) | -8.46 | 26.48 | -60.37 – 43.45 | -0.32 | 0.93 |

# **Table 3 Weighted ANCOVA table for COPD mesured with LLN and steps**

|  | **Estimate** | **SE** | **95% CI** | **t** | ***p*** |
| --- | --- | --- | --- | --- | --- |
| COPDLLN | 427.24 | 412.86 | -381.94 – 1236.43 | 1.04 | 0.3 |
| Age | -21.41 | 6.22 | -33.59 – -9.22 | -3.44 | 0.0006 |
| BMI | -88.75 | 15.13 | -118.41 – -59.09 | -5.86 | 0.0008E-5 |
| Sex (women) | -1005.72 | 144.44 | -1288.82 – -722.62 | -6.96 | 0.0001E-6 |
| Accelerometer wearing | 876.26 | 59.44 | 759.75 – 992.77 | 14.74 | 0.0002E-12 |
| Worked last year  Study/retired  Working | -110.33  727.81 | 319.23  221.81 | -736.01 – 515.36  -293.06 – 1162.55 | -0.35  3.28 | 0.73  0.001 |
| Seasons  Spring  Summer  Winter | 107.2  144.78  -824.54 | 271.36  239.6  234.37 | -424.66 – 639.06  -324.81 – 614.38  -1283.91 – -365.17 | 0.4  0.6  -3.52 | 0.69  0.55  0.0005 |
| Marital status (couple) | -237.41 | 203.6 | -636.46 – 161.64 | -1.17 | 0.24 |
| Levels of cotinine | -0.63 | 0.16 | -0.94 – -0.32 | -3.94 | 0.0009E-1 |
| Education  High school  Work school  College  University < Bachelor  Bachelor  University > Bachelor  Missing | 868.72  448.84  152.96  709.52  719.19  770.98  1074.54 | 398.92  394.08  365.73  337.95  417.7  456.44  364.78 | 86.85 – 1650.59  -323.54 – 1221.22  -563.85 – 869.77  47.15 – 1371.9  -99.49 – 1537.87  -123.62 – 1665.58  359.6 – 1789.49 | 2.18  1.14  0.42  2.1  1.72  1.69  2.95 | 0.03  0.26  0.68  0.04  0.09  0.09  0.003 |
| Household income  $15k-$19,99k  $20k-$29,99k  $30k-$39,99k  $40k-$49,99k  $50k-$59,99k  $60k-$79,99k  $80k-$99,99k  ≥ $100k | -702.44  -236.96  -224.68  -226.19  140.17  84.67  398.15  332.3 | 561.65  556.16  506.42  594.22  525.09  538.48  455.27  499.68 | -1803.25 – 398.36  -1327.02 – 853.1  -1217.24 – 767.88  -1390.84 – 938.45  -888.98 – 1169.32  -970.74 – 1140.08  -494.16 – 1290.47  -647.05 – 1311.65 | -1.25  -0.43  -0.44  -0.38  0.27  0.16  0.88  0.67 | 0.21  0.67  0.66  0.7  0.79  0.88  0.38  0.51 |
| ∆ (FEV_indv_-0.7) | -105.77 | 1375.61 | -2801.91 – 2590.38 | -0.08 | 0.94 |

# **Table 4 Weighted ANCOVA table for COPD mesured with LLN and sedentary behavior**

|  | **Estimate** | **SE** | **95% CI** | **t** | ***p*** |
| --- | --- | --- | --- | --- | --- |
| COPDLLN | -12.27 | 8.77 | -29.45 – 4.91 | -1.4 | 0.16 |
| Age | 1.84 | 0.17 | 1.51 – 2.17 | 10.92 | 0.0002E-12 |
| BMI | 0.4 | 0.34 | -0.27 – 1.07 | 1.18 | 0.24 |
| Sex (women) | 18.37 | 4.22 | 10.11 – 26.64 | 4.36 | 0.0002E-1 |
| Accelerometer wearing | 34.55 | 1.32 | 31.97 – 37.13 | 26.24 | 0.0002E-12 |
| Worked last year  Study/retired  Working | -5.08  -24.65 | 6.46  5.39 | -17.75 – 7.59  -35.21 – -14.1 | -0.79  -4.58 | 0.43  0.0006E-2 |
| Seasons  Spring  Summer  Winter | 0.47  -10.69  12.65 | 5.03  6.74  6.5 | -9.4 – 10.33  -23.9 – 2.53  -0.09 – 25.39 | 0.09  -1.59  1.95 | 0.93  0.11  0.05 |
| Marital status (couple) | 2.46 | 4.55 | -6.46 – 11.38 | 0.54 | 0.59 |
| Levels of cotinine | 0.003 | 0.004 | -0.004 – 0.01 | 0.82 | 0.41 |
| Education  High school  Work school  College  University < Bachelor  Bachelor  University > Bachelor  Missing | -18.15  7.06  6.22  17.27  12.98  12.96  -13.74 | 12.99  10.23  11.69  8.82  9.87  14.95  7.77 | -43.61 – 7.32  -12.99 – 27.11  -16.69 – 29.13  -0.02 – 34.56  -6.36 – 32.33  -16.33 – 42.26  -28.97 – 1.49 | -1.4  0.69  0.53  1.96  1.32  0.87  -1.77 | 0.16  0.49  0.59  0.05  0.19  0.39  0.08 |
| Household income  $15k-$19,99k  $20k-$29,99k  $30k-$39,99k  $40k-$49,99k  $50k-$59,99k  $60k-$79,99k  $80k-$99,99k  ≥ $100k | 9.94  5.84  -0.4  -6.31  3.43  -14.08  -9.17  -3.39 | 13.24  12.83  12.33  12.7  12.39  12.06  12.27  10.93 | -16.01 – 35.88  -19.31 – 30.98  -24.56 – 23.76  -31.19 – 18.57  -20.86 – 27.72  -37.71 – 9.55  -33.22 – 14.88  -24.81 – 18.03 | 0.75  0.46  -0.03  -0.5  0.28  -1.17  -0.75  -0.31 | 0.45  0.65  0.97  0.62  0.78  0.24  0.46  0.76 |
| ∆ (FEV_indv_-0.7) | -7.96 | 34.41 | -75.41 – 59.48 | -0.23 | 0.82 |
